# Comparative Genomic Analysis Confirms Five Genetic Populations of the Select Agent, *Rathayibacter toxicus*

**DOI:** 10.3390/microorganisms8030366

**Published:** 2020-03-05

**Authors:** Jarred Yasuhara-Bell, Mohammad Arif, Grethel Y. Busot, Rachel Mann, Brendan Rodoni, James P. Stack

**Affiliations:** 1 Department of Plant Pathology, Kansas State University, 1712 Claflin Road, 4024 Throckmorton Plant Science Center, Manhattan, KS 66506, USA; jarredyb@ksu.edu (J.Y.-B.); gybusot@gmail.com (G.Y.B.); 2 Plant Biosecurity Cooperative Research Centre, CRC for National Plant Biosecurity, Level 2, Building 22, Innovation Centre, University Drive, University of Canberra, Bruce, Australian Capital Territory, Canberra 2617, Australia; arif@hawaii.edu (M.A.); Rachel.Mann@ecodev.vic.gov.au (R.M.); Brendan.Rodoni@ecodev.vic.gov.au (B.R.); 3 Department of Plant and Environmental Protection Sciences, University of Hawai`i at Mānoa, Honolulu, HI 96822, USA; 4 Department of Jobs, Precincts and Regions, Microbial Sciences, Pests & Diseases, Agriculture Victoria, AgriBio Centre, La Trobe University, 5 Ring Rd, Bundoora, Victoria 3083, Australia

**Keywords:** *Rathayibacter toxicus*, annual ryegrass toxicity, flood plain staggers, Stuart’s range syndrome, comparative genomics, populations

## Abstract

*Rathayibacter toxicus* is a Gram-positive, nematode-vectored bacterium that infects several grass species in the family Poaceae. Unique in its genus, *R. toxicus* has the smallest genome, possesses a complete CRISPR-Cas system, a vancomycin-resistance cassette, produces tunicamycin, a corynetoxin responsible for livestock deaths in Australia, and is designated a Select Agent in the United States. In-depth, genome-wide analyses performed in this study support the previously designated five genetic populations, with a core genome comprising approximately 80% of the genome for all populations. Results varied as a function of the type of analysis and when using different bioinformatics tools for the same analysis; e.g., some programs failed to identify specific genomic regions that were actually present. The software variance highlights the need to verify bioinformatics results by additional methods; e.g., PCR, mapping genes to genomes, use of multiple algorithms). These analyses suggest the following relationships among populations: RT-IV ↔ RT-I ↔ RT-II ↔ RT-III ↔ RT-V, with RT-IV and RT-V being the most unrelated. This is the most comprehensive analysis of *R. toxicus* that included populations RT-I and RT-V. Future studies require underrepresented populations and more recent isolates from varied hosts and geographic locations.

## 1. Introduction

The genus *Rathayibacter* was described relatively recently [1] and now comprises nine species of Gram-positive plant-pathogenic bacteria, including *R. agropyri* [2,3], *R. caricis* [4], *R. festucae* [4], *R. iranicus* [1], *R. oskolensis* [5], *R. rathayi* [1], *R. tanaceti* [6], *R. toxicus* [7,8] and *R. tritici* [1]. *Rathayibacter toxicus* [7,8] infects several grass species in the family Poaceae [9], including annual ryegrass (*Lolium rigidum*) [10], annual beard grass (*Polypogon monspeliensis*) [11,12], and bent or blown grass (*Agrostis avenacea*) [12]. Like other *Rathayibacter* species, *R. toxicus* is vectored by several species of seed-gall nematodes in the genus *Anguina* [9,11,12,13,14,15]; the nematode vector determines the plant host that *R. toxicus* infects. During the disease cycle, *R. toxicus* can produce a tunicamycin-like corynetoxin [16,17,18,19,20] that inhibits cell-wall biosynthesis and interferes with protein glycosylation. It has been speculated that the toxin is used to kill the nematode and/or other microorganisms within the gall to reduce competition for resources [21]; however, it does cause devastating off-target effects when livestock and horses feed and/or graze on diseased plant material contaminated with the toxin [7,9,10,22]. Toxicoses result in fetal abortion in pregnant females, severe neurological and hepatic damage, and often death [17,23,24]. *Rathayibacter toxicus*-induced toxicities are known in Australia as annual ryegrass toxicity (ARGT), flood plain staggers and Stewart’s Range syndrome [7,10,13,17,25,26].

Both the bacterium and the nematode vector have the capability to survive several years in a desiccated state [9] and have evolved to reactivate in near synchrony with seed germination and seedling development of plant hosts [22]. The long survival potential of the pathogen and the vector in a desiccated state increases the concern for long-distance dissemination in seed and hay export products. Management of ARGT in Australia is achieved through management of the plant host, for example, early cutting prior to seed development, rather than directly targeting either the bacterium or the nematode vector [9,22]. Unfortunately, many of the grass hosts of *R. toxicus*, including annual ryegrass, become invasive and colonize areas adjacent to and rapidly spread from production sites. This increases the challenge of effective management and reduces the potential for successful eradication.

Presently, *R. toxicus* is limited geographically to Australia, being reported in Western Australia, South Australia and New South Wales [7,10,13], and potentially the Cape Province of South Africa [25,27]. *R. toxicus* has not been reported in the United States; however, the nematode vector and plant host species are present. Trade and travel pose an increased risk of dissemination of *R. toxicus* to non-endemic regions, both intra- and inter-continental. As a result, *R. toxicus* was designated as a Plant Pathogen Select Agent under seven CFR 331 by the U.S. Department of Agriculture (USDA) Animal and Plant Health Inspection Service (APHIS) in 2008 [21], due to potentially significant socioeconomic impacts resulting from mass livestock deaths.

Within the genus of *Rathayibacter*, *R. toxicus* is genetically unique from other species; it is the only species of *Rathayibacter* to possess clustered regularly interspaced short palindromic repeats (CRISPR) and CRISPR-associated proteins (Cas) as part of the CRISPR-Cas system [19,28,29,30], it is the only *Rathayibacter* species reported to contain a functional tunicamycin gene cluster [16,17,18,19,20], and it possesses the smallest genome with the lowest G+C content. Among *R. toxicus* isolates, sub-specific groups (populations) have been identified using several techniques, including amplified fragment-length polymorphisms (AFLP) analysis and pulse-field gel electrophoresis (PFGE), with Western Australian isolates (Group A; RT-III) being genetically distinct from those found in South Australia (Group B; RT-II), and one strain (FH100; Group C) grouping separately [31,32,33,34]. Recently, Arif et al. [35] used multi-locus sequence typing (MLST) and inter-simple sequence repeat (ISSR; inter-microsatellite) analysis and confirmed previous findings, as well as identified a newly emergent population in South Australia, designated RT-I, that was genetically distinct from both RT-II and RT-III. Davis II et al. [28,30] used whole-genome single nucleotide polymorphism (SNP) analysis and confirmed earlier groupings; however, this study did not include isolates from population RT-I. A recent study replicated the MLST analysis by Arif et al. [35] using additional isolates and described two additional genetic populations, RT-IV (based on two isolates from New South Wales, Australia) and RT-V (based on two isolates from southeast South Australia) [36]. The objective of the present study was to use a multifaceted approach, based on genome-wide analyses, to more completely characterize the *R. toxicus* genome and further investigate variation among *R. toxicus* populations. This study represents the first in-depth genome-wide investigation of *R. toxicus* to includes all five populations. Genomic data is presented in its entirety and results described in this study confirm the existence of five genetically distinct populations.

## 2. Materials and Methods

### 2.1. Genome Sequences

Whole-genome sequences (WGS) for *Rathayibacter toxicus* strains 70137 and WAC3373 were obtained from the National Center for Biotechnology Information (NCBI) [37] GenBank nucleotide database [38,39,40]. WGS for *R. toxicus* strains SA03-04 [41], SA19-14, WA40-23C, WAC7056 (type strain), CS28, CS36, CS38 and CS39 were obtained using PacBio RS II single molecule real-time (SMRT) sequencing (Pacific Biosciences, Menlo Park, CA, USA). WGS for *R. toxicus* strains SA03-14, SA03-19, SA08-07, SA08-08, SA08-09, SA19-02, SA19-06, SA19-07 were obtained using Illumina MiSeq (Illumina Inc., San Diego, CA, USA); PacBio sequencing data were de novo assembled with HGAP [42] using default parameters (500 bp min. subread length; 6 kb min. seed read length) and polished with Quiver. Illumina MiSeq data were assembled by mapping to complete PacBio genomes using Bowtie2 [43] in Geneious version 7.1.9 [44], and/or de novo using the Geneious assembler [44]. A single-contig complete genome was not obtained for CS38 and genome assembly for SA08-07 was not adequate; therefore, these two strains were excluded from whole-genome analyses. Sequence data for CS38 and SA08-07 were adequate for gene extraction and therefore included in specific-gene and multi-locus sequence analysis (MLSA). All single-contig complete genomes were reoriented with the replication initiation factor (*dnaA*) gene as the starting point, using MEGA7 [45,46]. PacBio sequence data for representative strains of each population group (strains SA03-04, WAC7056, WA40-23C, CS36 and CS39 for groups RT-I, RT-II, RT-III, RT-IV and RT-V, respectively) were annotated through the United States Department of Energy (US DOE) Joint Genome Institute (JGI) (https://img.jgi.doe.gov/cgi-bin/submit/main.cgi) Integrated Microbial Genome Expert Review (IGM/ER) using the Isolate Genome Gene Calling method (File S1; Figure S1). Information about the strains used in this study is presented in Table 1. Genome sequences were deposited into NCBI GenBank, with annotations created using the NCBI Prokaryotic Genome Annotation Pipeline (PGAP) [47,48] (File S2).

### 2.2. Genome Content

Rapid Annotation using Subsystem Technology (RAST) version 2.0 [49] (http://rast.nmpdr.org/) was used to determine gene content based on functional subsystem classifications (Files S3, S4). Data was accessed and viewed using the SEED Viewer version 2.0 [50]. tRNAscan-SE [51] (http://lowelab.ucsc.edu/tRNAscan-SE/) was used to determine the number and types of tRNAs, as well as predict pseudogene numbers. The following software tools with web-based interfaces were used to identify bacterial secretion systems: KEGG (Kyoto Encyclopedia of Genes and Genomes) [52,53,54,55] (https://www.genome.jp/kegg-bin/get_htext), TXSScan MacSyFinder [56,57] (https://galaxy.pasteur.fr/root?tool_id=toolshed.pasteur.fr/repos/odoppelt/txsscan/TXSScan/) in Galaxy [58,59,60], T346Hunter [61] (http://bacterial-virulence-factors.cbgp.upm.es/T346Hunter), SecReT4 [62] T4SS Location (http://db-mml.sjtu.edu.cn/SecReT4/) and SecReT6 [63] T6SS-HMMER [64] (http://db-mml.sjtu.edu.cn/SecReT6/). PanSeq [65] was used to analyze the genomes of each population to identify unique regions.

### 2.3. Average Nucleotide Identity

Average nucleotide identity (ANI) was determined using three independent software tools with available online web-servers: (1) the genome-based distance matrix calculator [66] (http://enve-omics.ce.gatech.edu/g), which estimates ANI using both best hits (one-way ANI) and reciprocal best hits (two-way ANI) [67]; (2) EZGenome [68] (https://www.ezbiocloud.net/tools/ani), which uses the OrthoANIu algorithm [69], which incorporates USEARCH [70] instead of BLAST (Basic Local Alignment Search Tool) [71] to estimate ANI; (3) JSpeciesWS [72] (http://jspecies.ribohost.com/jspeciesws/), which estimates ANI based on BLAST+ [73] (ANIb) [67] or MUMmer [74,75,76] (ANIm), as well as tetra-nucleotide signatures (Tetra) [77,78,79,80]. Heat maps were generated by inputting data into Heatmapper [81] (http://www2.heatmapper.ca/). The genome-based distance calculator tree output file was input into Geneious version 7.1.9 [44] to produce a phylogenetic tree.

### 2.4. In Silico DNA-DNA Hybridization

Digital DNA-DNA hybridization (dDDH) values were obtained using the Genome-to-Genome Distance Calculator (GGDC) version 2.1 [82,83,84] (http://ggdc.dsmz.de/). High-scoring segments pairs (HSP) were calculated using BLAST+ (the recommended default) [67,73], as well as MUMmer [74,75,76]. Genome-to-genome distance values were calculated according to three formulas: Formula 1—the length of all HSPs divided by total genome length; Formula 2—sum of all identities found in HSPs divided by overall HSP length; Formula 3—sum of all identities found in HSPs divided by total genome length. dDDH values corresponding to each distance formula were calculated based on generalized linear models (GLM) [85]. Differences in G+C content were also calculated [86]. Heat maps were generated by inputting data into Heatmapper [81] (http://www2.heatmapper.ca/).

### 2.5. Pangenome Analysis

The MicroScope Microbial Genome Annotation and Analysis Platform version 3.10.3 [87] (https://www.genoscope.cns.fr/agc/microscope/home/index.php) was used to determine the pan/core/variable-genome of representative strains of *R. toxicus* from each genetic population (File S5). MicroScope gene families (MICFAM) were computed using an algorithm implemented in SiLiX [88], under stringent parameters (80% amino-acid identity, 80% amino-acid alignment coverage). 

### 2.6. Identification of Prophage and Phage Remnants

Several programs were used to analyze genomes for prophage insertions and phage remnants, including PhageWeb [89] (http://computationalbiology.ufpa.br/phageweb/index.php), PHAST (PHAge Search Tool) [90] (http://phast.wishartlab.com/), PHASTER (PHAge Search Tool Enhanced Release) [91] (http://phaster.ca/), Prophinder [92] (http://aclame.ulb.ac.be/perl/Aclame/Prophages/prophinder.cgi?), and VirSorter version 1.0.3 [93] in the CyVerse Discovery Environment (https://de.cyverse.org/de/). Identified regions were extracted from respective genomes using Geneious version 7.1.9 [44] and analyzed using NCBI BLAST [37,71], ViroBLAST [94] (https://indra.mullins.microbiol.washington.edu/viroblast/viroblast.php), and the Actinobacteriophage Database [95] (https://phagesdb.org/blast/). 

### 2.7. Secondary Metabolite and Biosynthetic Gene Cluster Analysis

AntiSMASH 4.0 [96,97,98,99] (https://antismash.secondarymetabolites.org/#!/start) was used to identify secondary metabolite BGCs (Files S6–S7) and cluster BLAST [71] results were analyzed. 

### 2.8. Horizontal Gene Transfer

IslandViewer4 [100,101] (https://www.pathogenomics.sfu.ca/islandviewer/upload/), using IslandPick [102], IslandPath-DIMOB [103,104] and SIGI-HMM [105,106], was used to identify putative HGT events in representative strains of *R. toxicus*. The RGP (Region of Genome Plasticity) Finder tool in MicroScope Microbial Genome Annotation and Analysis Platform version 3.10.3 [87] (https://www.genoscope.cns.fr/agc/microscope/compgenomics/genomicIsland.php) was also used to identify horizontally transferred genes in representative strains of *R. toxicus*. MicroScope also employs AlienHunter interpolated variable order motifs (IVOM) [107] and SIGI-HMM [105,106] to identify potential horizontal gene transfer events.

### 2.9. Individual Gene, Gene Cluster, Multi-Locus and Whole-Genome Sequence Analyses

MAUVE version 2.4.0 [108] was used to perform full genome sequence alignments with the progressive Mauve algorithm. The MAUVE guide tree output file was input into Geneious version 7.1.9 [44] to produce a phylogenetic tree.

The Reference sequence Alignment based Phylogeny builder (REALPHY) [109] version 1.12 (https://realphy.unibas.ch/realphy/) was used to infer phylogenetic trees from whole-genome sequence data. All sequences were mapped to a provided reference sequence (SA03-04) via Bowtie2 [43] and phylogenetic trees inferred via PhyML (PHYlogenetic inferences using Maximum Likelihood) [110,111]. REALPHY alignment outputs were also used as inputs into the Geneious version 7.1.9 [44] to produce a neighbor-joining (NJ) tree [112] constructed using the Jukes-Cantor model [113]. Confidence intervals were assessed using the bootstrap method with 1000 replications. [114].

PhyloSift [115] analysis was performed on all complete *R. toxicus* genome sequences. Sequences were queried for similarities to genes contained in a reference database using LAST [116]. The hmmalign program from the HMMER 3.0 software package [64] was used to concatenate and align marker gene sequences. The Phylogenetic Placer (pplacer) program [117] was used to infer phylogenies and produce a phylogenetic tree under default conditions (Maximum-likelihood). The output tree file was input into FigTree v1.4.3 (tree.bio.ed.ac.uk/software/figtree/) to produce the phylogenetic tree.

Several genes and gene clusters were analyzed in this study, which include: exopolysaccharide (EPS) production protein; vancomycin resistance genes (*vanH*—d-lactate dehydrogenase; *vanA*—d-alanine-d-alanine ligase; *vanX*—d-alanyl-d-alanine depeptidase); TPS-TPP and TreY-TreZ trehalose biosynthetic pathway genes (*tps*—trehalose-6-phosphate synthase; *tpp*—trehalose-6-phosphate phosphatase; *treY*—maltooligosyl-trehalose synthase; *treZ*—maltooligosyl-trehalose trehalohydrolase); tunicamycin biosynthesis genes (*tunA*—dTDP-glucose 4,6-dehydratase/UDP-glucose 4-epimerase; *tunB*—MoaA/NifB/PqqE/SkfB family radical SAM enzyme; *tunC*—N-Acyltransferase; *tunD*—Glycosyl transferase family 1; *tunE*—LmbE family N-acetylglucosaminyl deacetylase; *tunF*—UDP-glucose/galactose 4-epimerase; *tunG*—broad specificity UMP phosphatase PhoE; *tunH*—type I phosphodiesterase/UDP-tunicaminyluracil pyrophosphatase; *tunI*—ABC-2 type transport system ABC-binding subunit; *tunJ*—ABC-2 type transport system permease subunit; *tunK*—Acyl-carrier protein; *tunL*—3-oxoacyl-[acyl-carrier-protein] synthase II); ATP synthase β chain (*atpD*); heat shock protein 70 (*dnaK*); DNA gyrase (topoisomerase II) β subunit (*gyrB*); polyphosphate kinase (*ppK*); recombinase A (*recA*); DNA-directed RNA polymerase β chain (*rpoB*). The latter six genes were used for MLSA. Gene sequences were extracted from WGS using Geneious version 7.1.9 [44]. Individual gene sequences were aligned using ClustalW [118] for sequences <2000 nt and MAFFT (multiple sequence alignment based on fast Fourier transform) [119] for those >2000 nt. Complete gene sequences were also translated into amino acid sequences and aligned using the block substitution matrix (Blosum62) [120]. Genes belonging to gene clusters were also analyzed by concatenating gene sequences prior to alignment. MLSA involved concatenating all six genes in the order listed previously, followed by alignment. Neighbor-joining trees [112] were constructed using the Jukes-Cantor model [113]. Confidence intervals were assessed using the bootstrap method with 1000 replications. [114]. NCBI BLAST analyses [37,71] were performed on all gene sequences mentioned previously to identify potential homologs in other organisms. Genes and gene clusters examined in this study were also mapped to their respective genomes using Bowtie2 [43] in Geneious version 7.1.9 [44].

Genes with potentially interesting annotations (e.g., potential phage-related, virulence, and/or protection/defense genes) were also analyzed. These include: kojibiose/trehalose phosphorylase (2505 nt); sucrose synthase/glycosyltransferase involved in cell wall biosynthesis (2133 nt); capsular EPS synthesis protein (1461 nt); cyclic-di-GMP-binding biofilm dispersal mediator protein (675 nt); EPS phosphotransferase CpsY/stealth-like protein (1584 nt); cell division trigger factor (1392 nt); vancomycin resistance protein VanJ (897 nt) (separate from the *vanHAX* cluster); phage-shock protein PspC (300 nt); cell division inhibitor protein (513 nt); cell division-specific peptidoglycan biosynthesis regulator (1278 nt); type-III secretion system (T3SS) inner membrane Yop/YscD-like protein (492 nt); nucleotidyltransferase AbiEii toxin of the type-IV toxin-antitoxin system (987 nt); T3SS SseB-like protein (6783 nt); cell division inhibitor protein (1059 nt); zeta toxin (948 nt); phage-related protein SprT-like (462 nt); prophage Lp2 protein 6 (1092 nt). Gene nucleotide sequences were analyzed individually and in an MLSA.

## 3. Results and Discussion

### 3.1. Genome Content and Organization

RAST (Rapid Annotation using Subsystem Technology) and JGI (Joint Genome Institute) analyses. Single-contig WGS were analyzed using RAST. A summary of general strain comparisons by RAST is shown in Table S1. RAST determined all RT-I strains possess 314 subsystems, with the exception of strain SA19-07 (315 subsystems), thus demonstrating the potential for variation even within populations. RT-II and RT-III both possess 315 subsystems, while RT-IV and RT-V possess 314 and 311 subsystems, respectively. A simple analysis of the subsystems between populations would suggest that RT-I is most similar to RT-IV, as is RT-II and RT-III, based on number and types of subsystems. RAST results show all *R. toxicus* strains code for 51 RNAs. A summary of the number of subsystem features identified by RAST is shown in Table S2. 

Strains SA03-04, WAC7056, WA40-23C, CS36 and CS39 were chosen to represent populations RT-I, RT-II, RT-III, RT-IV and RT-V, respectively, and submitted to JGI for comprehensive annotation. These strains were chosen because single-contig whole-genome sequences were obtained with PacBio. Strain WA40-23C was chosen to represent RT-III as it is the most recent isolate and thought to represent the current RT-III population in Western Australia. A summary of general strain comparisons by JGI is shown in Table S3. JGI results show that all the representative strains possess 51 RNAs (six rRNAs and 45 tRNAs) and five other RNAs. tRNAscan-SE showed that all *R. toxicus* strains code for 45 tRNAs, comprising 41 tRNAs for standard amino acids and four tRNAs to mismatch isotypes (Table S4). RAST, JGI and tRNAscan-SE data were congruent.

To produce a comprehensive annotation, JGI used multiple databases to make gene calls, including KEGG [52,53,54,55], COG (Clusters of Orthologous Groups) [121,122,123,124], KOG (EuKaryotic Orthologous Groups) [125], Pfam (Protein Families Database) [126,127,128], and TIGRfam (The Institute for Genomic Research) [129,130,131,132]. KEGG analysis compared representative strains based on 35 functional categories (Table S5), many of which typically would not apply to prokaryotes. TIGRfam analysis compared representative strains based on 17 functional categories (Table S6), with one category being attributed to unknown functions. The functional categories produced by KEGG and TIGRfam were different from each other as well as those produced by RAST, precluding direct comparisons. COG, KOG and Pfam have most of the same functional categories, and thus allowed comparison. COG, KOG and Pfam analysis compared representative strains based on 23, 21 and 19 functional categories (Tables S7–S9), respectively, with two categories being attributed to unknown functions and general function prediction only. The functional categories of COG, KOG and Pfam, while common to all, differed in the counts attributed to each category across each database. 

Results demonstrate that depending on the programs and/or databases used to analyze genomes, varying gene counts, gene locations, and functional annotations can be obtained. JGI was chosen for analysis because it considers multiple databases to make gene calls for annotation, thereby increasing its accuracy. However, both JGI and RAST did not identify the tunicamycin biosynthetic gene cluster as part of any functional category or pathway, particularly regarding secondary metabolite biosynthesis. Combined RAST and JGI data suggest both inter- and intra-population variations exist; however, no major differences were observed between any strain for a given category, suggesting that in silico analysis of gene function may not be the most effective tool for discerning populations of *R. toxicus*. It appears necessary to use multiple programs and/or databases and then create a consensus annotation to more accurately represent the gene content within an organism’s genome. To account for this, RAST, PGAP and JGI annotations were used together in subsequent analyses.

Secondary metabolite and biosynthetic gene clusters. AntiSMASH 4.0 identified 20–22 BGCs among all strains of *R. toxicus* tested (Table S10), with the exception of RT-I strain SA03-14. The 22 AntiSMASH clusters (ASCs) are shown in Table S11, including tunicamycin biosynthesis. Twenty-one ASCs were identified in RT-I, RT-III and RT-IV populations, except for RT-I strain SA03-14, for which AntiSMASH identified only seven. The 14 unidentified ASCs in SA03-14 were found after mapping the ASCs from SA03-04 to the genome sequence of SA03-14 using Bowtie2 in Geneious, demonstrating that these BGCs are actually present in strain SA03-14. It is unknown why AntiSMASH failed to identify these BGCs in strain SA03-14; perhaps the algorithm used to identify BGCs failed due to some sequence variation, either inherent to the strain or as a result of sequencing error. Twenty ASCs were identified in the RT-II population, with strains missing ASC-14 (phosphonate BGC). Twenty-two ASCs were identified in RT-V, which include a thiopeptide BGC unique to this population. A summary of the gene function/content for BGCs identified by AntiSMASH is in File S8. It is interesting to note that the clusters showed size variation among the strains tested, even though the function of the BGCs common to all strains were identified as being the same (Figure S2). The prime example is ASC-2 in strain CS39, which was much larger than ASC-2 in all other strains and extended into the CRISPR-Cas portion of the genome (Figure S2D). The reason for these differences is unknown.

It is of note to mention that reanalysis with the new AntiSMASH 4.2.0 yielded different results; AntiSMASH identified only seven BGCs in RT-I, RT-II and RT-III and eight in RT-IV and RT-V. RT-I and RT-III contained the same seven ASCs: T3PKS (type-III polyketide synthase) (pactamycin BGC); Other (BD-12 BGC); Nucleoside-Asylpolyene-Nrps (nonribosomal peptide synthetase) (tunicamycin BGC); Lantipeptide; Phosphonate; Phosphonate-Thiopeptide (rhizocticin BGC); Terpene (Carotenoid BGC). RT-II did not possess the Phophonate BGC but had an Nrps (dynemicin BGC) between the Lantipeptide and Phosphonate-Thiopeptide BGCs. RT-IV contained all eight of these BGCs, with the Nrps (Dynemicin BGC) being between the Phosphonate and Phosphonate-Thiopeptide BGCs. RT-V also had eight BGCs; however, RT-V did not contain the Lantipeptide BGC and had a unique Thiopeptide BGC that was between the Nrps (dynemicin BGC) and Phosphonate-Thiopeptide BGCs. All strains of a given population had the same gene clusters, thus further validating the population groupings; new analysis with AntiSMASH 4.2.0 did not show anomalies in strain SA03-14 as with AntiSMASH 4.0; the reason for the large discrepancy in number of BGCs identified by AntiSMASH 4.0 relative to AntiSMASH 4.2.0 is unknown, but likely due to a major change in the search algorithm.

Horizontal gene transfer. JGI analysis identifies putative genes suggested to have been acquired by an HGT event (Table S12). A total of 15, 16 and 7 genes were identified for representative strains of populations RT-I, RT-IV and RT-V, respectively, with zero being identified for both RT-II and RT-III. This suggests that RT-II and RT-III are most closely related. Twelve of the genes identified in RT-I and RT-IV are identical between these two populations and not present in RT-V, suggesting that these two populations are most closely related. JGI HGT results for population RT-V identified a thiopeptide-type bacteriocin biosynthesis protein, which was suggested to have come from *Streptomyces* sp. This gene is part of ASC-18, the thiopeptide cluster identified by AntiSMASH to be unique to RT-V.

IslandViewer4, which incorporates IslandPick, IslandPath-DIMOB and SIGI-HMM to find potential HGT events, was used to analyze five strains acting as representatives for each of the five population of *R. toxicus*. IslandPick identified only one region in both RT-I and RT-IV (Table S13; Figure S3). IslandPath-DIMOB identified 15, 14, 15, 15 and 14 regions, while SIGI-HMM identified 6, 4, 4, 5 and 5 regions, in RT-I, RT-II, RT-III, RT-IV and RT-V, respectively; most regions found by either IslandPath-DIMOB or SIGI-HMM were the same, and most of the SIGI-HMM regions overlapped with those found by IslandPath-DIMOB (Table S13; Figure S3). 

The RGP Finder tool in MicroScope was used to identify unique regions in representative strains of *R. toxicus*. MicroScope, using AlienHunter IVOM and SIGI-HMM, was also used to identify potential HGT events. MicroScope identified 4, 3, 4, 6 and 3 RGPs in RT-I, RT-II, RT-III, RT-IV and RT-V, respectively (Table S13; Figure S2). SIGI-HMM in MicroScope identified 15, 17, 13, 15 and 15 regions, while IVOM identified 51, 55, 47, 53 and 47 regions, in RT-I, RT-II, RT-III, RT-IV and RT-V, respectively (Table S13; Figure S2). In RT-I, 12 SIGI-HMM and IVOM regions overlapped, but not with an RGP; RGP-1 and RGP-4 overlapped with both a SIGI-HMM and IVOM region, while RGP-2 and RGP-3 overlapped with only an IVOM. In RT-II, 15 SIGI-HMM and IVOM regions overlapped, but not with an RGP; RGP-1 and RGP-2 overlapped with both a SIGI-HMM and IVOM region, while RGP-3 overlapped with only an IVOM. In RT-III, 11 SIGI-HMM and IVOM regions overlapped, but not with an RGP; RGP-2 overlapped with both a SIGI-HMM and IVOM region, while RGP-1, RGP-3 and RGP-4 overlapped with only an IVOM. In RT-IV, 12 SIGI-HMM and IVOM regions overlapped, but not with an RGP; RGP-4 and RGP-6 overlapped with both a SIGI-HMM and IVOM region, while RGP-1, RGP-2, RGP-3 and RGP-5 overlapped with only an IVOM. In RT-V, 12 SIGI-HMM and IVOM regions overlapped, but not with an RGP; RGP-1 overlapped with both a SIGI-HMM and IVOM region, while RGP-2 and RGP-3 overlapped with only an IVOM.

In all, HGT regions were identified throughout the length of the genome (Figure S2). Interestingly, even though both IslandViewer and MicroScope use SIGI-HMM to identify HGT regions, there were a few exceptions that did not agree with each other; however, the majority of the SIGI-HMM regions overlapped between programs. Additionally, there was no instance in which all means for HGT identification agreed; there was no region that was identified by IslandPick, SIGI-HMM, IslandPath-DIMOB and IVOM. There was a variable overlap between the programs used, with some regions being identified by only a single program (Figure S2). It is of note that the CRISPR-Cas system and tunicamycin BGC are known to have been acquired through an HGT event. While the tunicamycin cluster (ASC-12) was identified by SIGI-HMM from both IslandViewer and MicroScope, as well as IVOM, neither IslandPick nor IslandPath-DIMOB identified this region. This was also observed for the majority of the CRISPR region; however, no regions were assigned to the Cas portion. Regions of suspected HGT were assigned to almost all ASCs, with slightly varying results depending on the length of a specific ASC in a specific strain; no HGT region was identified in ASC-5, ASC-7, ASC-8 and ASC-11.

IslandPick uses a comparative genomics approach to determine HGT events [102]. SIGI-HMM looks for codon bias using hidden Markov models (HMM) [105,106]. IslandPath-DIMOB looks for regions of at least eight genes, one of which has to be a mobility gene (transposase, integrase, etc.), that also has biased dinucleotide composition [103,104]. AlienHunter IVOM looks for compositional biases based on variable order motif distributions [107]. Lack of consensus among these programs reflects variations among the methods used and raises questions as to the accuracy of HGT event calling, especially since no single region was identified by all methods. Perhaps any significant HGT events happened so long ago that they have lost the majority of the defining factors, making identification difficult.

Prophage and phage remnants. PhageWeb, PHAST (PHAge Search Tool), PHASTER (PHAge Search Tool Enhanced Release), Prophinder, and VirSorter was used to identify potential prophage and phage remnants within the genome. PhageWeb, which compares sequence similarity to a phage database and considers changes in G+C and presence of flanking tRNA, did not identify any regions of potential phage origin. PHAST was able to identify a ~7 kb region. This region was considered incomplete by PHAST and had a score of 20. PHAST determined that this region contained seven proteins, six of which had matches to a phage protein database. These phage genes were considered to be similar to genes found in six phage species. These genes include a conserved protein of unknown function, a heme oxygenase, a PIF1-like helicase, an ABC-2 type transport permease, a copper transporter/copper resistance protein D, and a DNA-binding protein HU-1/HU-beta. Initially, analysis was performed on strains SA03-04, WAC7056, WA40-23C, CS36 and CS39, which were representative strains of RT-I, RT-II, RT-III, RT-IV and RT-V, respectively. The same ~7 kb region was identified in all strains, except WAC7056; it is yet unknown why PHAST did not identify this region in the representative RT-II strain. As a result, this region was mapped to the genome of WAC7056 using the Bowtie2 in Geneious and then extracted. Mapping revealed that the PHAST phage region flanks ASC-20 on the right (Figure S2). This process was repeated in all 18 strains of *R. toxicus* used in this study. Extracted sequences were aligned (7019 nt) using MAFFT and NJ trees were constructed using the Jukes-Cantor model, with confidence intervals being assessed using the bootstrap method with 1000 replications (Figure S4A). Relative to CS36 (RT-IV), RT-I contained seven unique SNPs and possessed a 12-nt deletion, RT-II and RT-III had three identical SNPs that were unique to these populations, and RT-V had one unique SNP. Phylogeny of this region would seem to indicate that RT-II and RT-III are one population that is more closely related to both RT-IV and RT-V, with RT-I being the most distant; however, analysis was based only on a few SNPs and the deletion of 12 nt in all RT-I strains relative to the other populations would account for it being the most distantly related. It is interesting to note that PHASTER, which is supposed to have a very similar phage identification pipeline as PHAST with exception of some software and hardware enhancements, did not identify any putative prophage regions within any of the genomes analyzed. Why this “enhanced” version of PHAST, which found a ~7 kb region, did not identify any regions remains unknown.

Prophinder, which compares query sequences to the ACLAME (a CLAssification of Mobile genetic Elements) database [133,134], was able to identify a different ~10 kb region that mapped to ASC-8 (Figure S1). This region contained an ABC-2 type transport system permease / O-antigen export system permease, a conserved protein of unknown function, a glycosyl-transferase involved in cell-wall biosynthesis, a group 1 glycosyl-transferase, a GDP-mannose 4,6-dehydrogenase, a GDP-mannose 4,6-dehydrogenase NAD(P)-binding subunit, a conserved protein of unknown function, an EPS production protein, and an acyl-CoA dehydrogenase / glutaryl-CoA dehydrogenase. Prophinder only provided results for CS36 (RT-IV) and WAC7056 (RT-II); it is yet unknown why it did not identify this region in other representative strains. As a result, this region was mapped to the genomes of all 18 strains of *R. toxicus* used in this study. Extracted sequences were aligned (10,128 nt) using MAFFT and NJ trees were constructed using the Jukes–Cantor model, with confidence intervals being assessed using the bootstrap method with 1000 replications (Figure S4B). Relative to RT-III, RT-I strains possessed 5 unique SNPs and one SNP in common with CS36 at position 5603. RT-II strains possessed unique SNPs. RT-IV possessed 15 unique SNPs and 7 SNPs in common with RT-V. RT-V possessed four unique SNPs. Phylogeny based on this region would suggest that RT-IV and RT-V are most closely related, followed by RT-I, then RT-III and RT-II; however, while this Prophinder region was larger than the PHAST region, it contained only slightly more SNPs.

VirSorter compares sequences to a database using HMM and BLAST and then detects viral regions by looking for the presence of viral “hallmark” genes, enrichment in viral, uncharacterized and/or short genes, depletion of Pfam affiliated genes, and depletion in strand switch. VirSorter was able to identify regions of varying size (~27–40 kb) in each of the representative *R. toxicus* strains. These regions were categorized as “category 3,” which means they possess low confidence. These regions were also identified as ambiguous, as they only present secondary viral metrics, but do not possess viral enrichment nor viral “hallmark” genes. Nevertheless, these regions were analyzed further. The regions varied in size but were all located in the same area and had overlapping sections; therefore, all regions were extracted and aligned in Geneious and used to produce consensus sequence. The consensus sequence was mapped to all 18 *R. toxicus* strains used in this study. Sequences were aligned (47,107 nt) using MAFFT and NJ trees were constructed using the Jukes-Cantor model, with confidence intervals being assessed using the bootstrap method with 1000 replications (Figure S4C). Phylogeny based on this region suggests that RT-IV and RT-V are most closely related, followed by RT-III, RT-II, and then RT-I.

In addition to all previous analyses, identified regions that were extracted from the representative genomes were also analyzed using NCBI BLAST, ViroBLAST, and the Actinobacteriophage Database. All three programs were unable to yield any usable results, as any hits to a phage genome were based on matches to only ~20–30 nt, suggesting these regions may not be of viral origin. The relationships established by all three phage regions differ from each other, as well as from other analyses. The arbitrary nature of the VirSorter region, along with the few numbers of SNPs compared in the other two analyses would suggest a poor indication of evolutionary relationships; however, together, all these analyses reinforce the existence of five distinct genetic populations. All three programs identified different regions of the genome as being of viral origin and the lack of concordance in calling a region as being of viral origin speaks to the differences in approaches used by each method. The fact that none of these methods identified the same region, or at least regions within the same vicinity, as well as the fact that one program yielded no results when a similar version of the program yielded results, points to vastly different methodologies with varying levels of accuracy. It is unknown if these regions are artifacts or true viral elements, but these discrepancies cast some doubt as to the authenticity of these designations. Regardless, the programs found these elements in all five populations of *R. toxicus*; therefore, if these are true prophage regions, this would suggest that they were acquired a long time ago, prior to the delineation of these populations. Perhaps all viral elements in *R. toxicus* were acquired a sufficient time ago that they lost their viral signatures and are now hard to differentiate from the rest of the genome. 

Secretion systems. T346Hunter, which uses HMMER [64] to identify type-III, type-IV and type-VI secretion systems (T3SS, T4SS and T6SS), found no T3SS/T4SS/T6SS clusters. SecReT4 and SecReT6, which use both HMMER3 [64] and Glimmer3 [135,136,137], were unable to find T4SS and T6SS clusters, respectively; however, some individual genes associated with secretion systems were identified. SecReT4 identified six individual genes associated with T6SS, including one copy each of *prgK* (multidomain peptidoglycan/murein hydrolase) [138], *tcpG* (cell-wall binding protein; peptidoglycan hydrolase) [139], *trbB* (P-type conjugative transfer ATPase; type-F conjugative transfer system pilin assembly thiol-disulfide isomerase) [140], *tcpA* (DNA segregation ATPase FtsK/SpoIIIE) [141,142,143] and *ofr14_Tn1* (NLP/P60 family lipoprotein), and three copies of traI_F (recombinase D; DNA helicase; relaxase) [144], in all representative strains of the five *R. toxicus* populations. Analysis of *R. tritici* strain NCPPB 1953 produced the same results as those for *R. toxicus*, except only two copies of *traI_F* were identified. SecReT6 found three copies of the T4SS *tssH* gene [145], an ATP-dependent Clp protease ATP-binding subunit, in all representative strains of the five *R. toxicus* populations. In contrast, analysis of the *R. tritici* strain NCPPB 1953 (GenBank accession CP015515.1) genome revealed five copies of *tssH*, as well as *tssP*, a polycystic kidney disease (PKD) repeat-containing protein. TXSScan also used HMMER3 [64] to identify flagellin, type-IV pili, tight adherence (Tad), T1SS, T2SS, T3SS, T4SS (types B, C, F, G, I and T), pT4SSi, pT4SSt, T5SS (a, b and c type), T6SS (types i, ii and iii), and T9SS genes. *virB6* (polytopic inner membrane essential for substrate secretion) [146,147,148] of the T4SS type-T was the only gene identified in all representative strains of the five *R. toxicus* populations, except RT-IV strain CS36, and the *R. tritici* strain NCPPB 1953 genome. TXSScan identified 3070, 2999, 3001, 3048 and 1806 *virB6* domains within the genomes of SA03-04 (RT-I), WAC7056 (RT-II), WA40-23C (RT-III), CS39 (RT-V) and *R. tritici* strain NCPPB 1953, respectively. It is unknown why *virB6* domains were absent from CS36 (RT-IV). The KEGG database only identified genes for the Sec and Tat pathways, which corroborates results obtained by the other software tools. The absence of any bacterial type secretion system is not surprising, as *R. toxicus* is a Gram-positive bacterium, which do not typically possess these advanced secretion systems.

**Figure 1:**
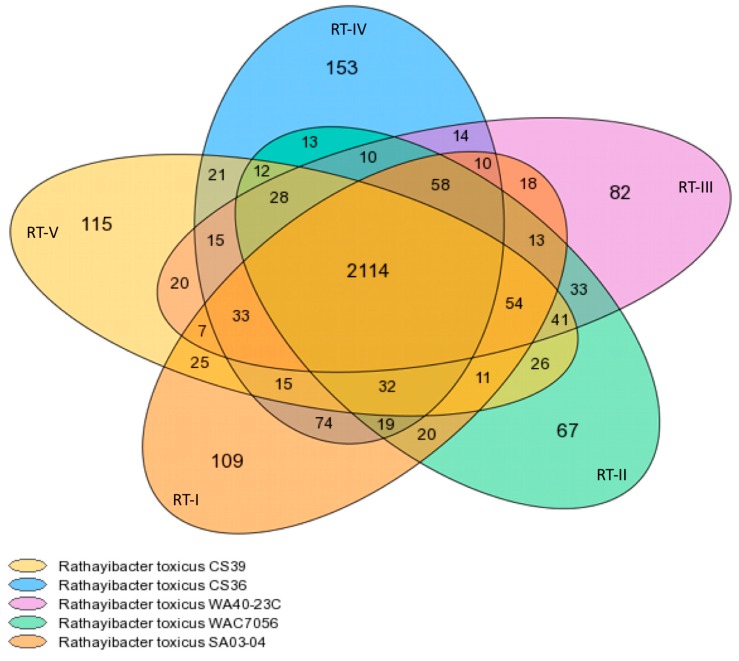
Core-/pan-genome for strains representing *R. toxicus* populations RT-I, RT-II, RT-III, RT-IV and RT-V. The MicroScope Microbial Genome Annotation and Analysis Platform was used to determine and compare the core-/pan-genome of *R. toxicus* populations. The numbers represent gene families, as designated by MicroScope. The center of the diagram represents the core-genome. The overlapping regions comprise the variable-genome and illustrate shared gene families among populations. The outer non-overlapping regions represent strain-(population-), specific gene families.

Core/Variable/Pan-genome analyses. The representative strains of *R. toxicus* populations were analyzed with the MicroScope Microbial Genome Annotation and Analysis Platform. The pan-, core-, and variable-genome for the five *R. toxicus* populations comprises 3262, 2114, and 1148 gene families (Figure 1), which correspond to 13,117, 10,812 and 2365 genes, respectively. The MicroScope summary output is shown in Table 2. Gene family counts (Table 2) differed slightly from those reported in the Venn diagram generated from the same program (Figure 1); the reason is unclear. Based on Figure 1, RT-I and RT-IV are most closely related, sharing the most total gene families (241) outside the core and having the most gene families shared between only these two populations (74). RT-I is next closest to RT-II and then RT-III, sharing 207 and 193 total gene families outside the core, and 20 and 18 gene families only between the two populations, respectively. RT-II and RT-III appear most closely related, sharing 237 total gene families outside the core and 33 gene families only between these two populations. RT-V is closest to RT-II, RT-III, and then RT-I, sharing 204, 198 and 177 total gene families outside the core, and 26, 20 and 25 gene families only between the two populations, respectively. RT-IV and RT-V were the least related, sharing only 156 total gene families outside the core and 21 gene families only between these two populations. RT-IV is closer to RT-II and then RT-III, sharing 172 and 168 total gene families outside the core, and 13 and 14 gene families only between the two populations, respectively. It is important to note that these numbers reflect gene families, as designated by MicroScope; the actual number of genes common between populations will differ, but the trends should remain the same. Based on shared gene family content, the relationship among strains is as follows: RT-IV ↔ RT-I ↔ RT-II ↔ RT-III ↔ RT-V. Unfortunately, evolutionary relationships cannot be determined based on gene content information only.

Annotations from MicroScope were compared to RAST, PGAP and JGI data, then consensus annotations were input manually. There were varying degrees of agreement between annotations, which differed in start-end/gene size and functional call, particularly in hypothetical genes. The core-genome data output was analyzed by RAST (Table S14). As expected, majority of the subsystems identified by RAST were located within the core-genome (97.46%–98.71%). Analysis of the core-genome by RAST only identified 10 RNAs. The MicroScope output only includes coding genes that could be assigned to functional gene families, therefore non-coding regions that may code for RNAs were not included; the 10 identified RNAs are most likely located within the sequence that also codes for a protein. The core-genome data was compiled and compared among the strains representing the five populations of *R. toxicus* (File S9). Consensus annotation resulted in ~27% of genes still having unknown functions.

By definition, the core-genome should only include sequence (gene content) contained in all genomes analyzed. In addition to genes essential for survival and basic functioning, other genes such as the CRISPR-Cas system, tunicamycin BGC, and vancomycin resistance were included within the core. Interestingly, the MicroScope program core outputs were not all the same among the strains analyzed. Certain genes were called in some strains and not others, which would suggest that they should be part of the variable genome; however, this occurred mainly with genes of unknown function. Additionally, some genes that were present in all strains were annotated as “fragments” in some strains relative to others. Even though some genes in the core-genome appear variable, it is believed that the sequences that these genes belong to are common among all strains, but the algorithm used makes different/extra gene calls based on sequence variation.

AntiSMASH identified 20 BGCs common to all strains tested, except for ASC-14 and ASC18, which was absent from RT-II and unique to RT-V, respectively. The majority of the genes included in these 20 ASCs are found in the core-genome. Even though ASC-14 was not identified in RT-II, genes from ASC-14 are present in the core-genome. However, ~12,000 nt from the middle of ASC-14 are not present in the core, suggesting this portion is missing from RT-II, which may be the reason why AntiSMASH could not identify it in RT-II. Additionally, ASC-18 is unique to RT-V (CS39), though the first 5 genes in this cluster appear in the core-genome.

The variable-genome data was compiled and compared among the strains representing the five populations of *R. toxicus* (File S10). A small subset of genes in the variable-genome could not be compiled due to various reasons; for example, for a given gene family designation, the products differed in length, some strains had an extra gene called proximal or distant to each other, possibly resulting from gene duplications, or a gene in one strain was in a completely different location, possibly resulting from a rearrangement. Consensus annotation resulted in ~79% of genes still having unknown function. Genes from twenty-one ASCs, except for ASC-18 which is unique to RT-V (CS39), were represented in the variable-genome to varying degrees. 

The strain-specific-genome data output (File S11) was also analyzed by RAST (Table S14). Only one subsystem was identified by RAST, which occurred in RT-V (CS39), and only 1 RNA was identified each in RT-I (SA03-04) and RT-IV (CS36). Consensus annotation resulted in ~90%, ~94%, ~76%, ~83% and ~84% of genes still having unknown functions for RT-I, RT-II, RT-III, RT-IV and RT-V, respectively. Genes from 10 ASCs (ASC-4, ASC-8, ASC-9, ASC-10, ASC-13, ASC-14, ASC-15, ASC-16, ASC-21, ASC-22) were represented in the RT-I-specific-genome to varying degrees. Genes from eight ASCs (ASC-4, ASC-8, ASC-9, ASC-10, ASC-13, ASC-15, ASC-19, ASC-20) were represented in the RT-II-specific-genome to varying degrees. Genes from nine ASCs (ASC-2, ASC-3, ASC-4, ASC-5, ASC-6, ASC-10, ASC-12, ASC-13, ASC-15) were represented in the RT-III-specific-genome to varying degrees. Genes from 13 ASCs (ASC-2, ASC-3, ASC-4, ASC-6, ASC-8, ASC-10, ASC-13, ASC-14, ASC-15, ASC-16, ASC-17, ASC-19, ASC-20) were represented in the RT-IV-specific-genome to varying degrees. Genes from 14 ASCs (ASC-2, ASC-3, ASC-4, ASC-7, ASC-9, ASC-13, ASC-14, ASC-15, ASC-16, ASC-17, ASC-18, ASC-19, ASC-20, ASC-21), including the unique thiopeptide cluster, were represented in the RT-V-specific-genome to varying degrees. It is of note to mention that MicroScope identified several proteins of unknown function in both the variable- and strain-specific-genome that were located within the CRISPR-Cas region of the genome. It is unclear how many inappropriate gene calls were made within the pan-genome or how this is reflected in the gene family/gene number comparisons; the numbers reported by MicroScope are likely higher than what actually occurs. 

PanSeq was used to query each population, relative to the others, to find regions unique to each population (Table 3). PanSeq identified 10, 6, 8, 10 and 4 regions for RT-I, RT-II, RT-III, RT-IV and RT-V, respectively, that vary in length from approximately 600–20,000 bp. All regions were aligned to strains from all populations using Geneious, which found several regions that exist in one or more populations. Results were corroborated when compared to the variable- and strain-specific-genome results, as well as when compared to AntiSMASH results. One example is that RT-III-5, RT-III-6 and RT-III-7 were found in all populations except RT-II. It was also found that these regions were located in ASC-14, which was not found in population RT-II, thus corroborating results. Another example is that three of the four regions found specific for RT-V were found to occur within ASC-18, which is a thiopeptide cluster unique to RT-V. With regards to the variable-genome, one example is that region RT-I-3 was identified in the variable-genome and genes attributed to this region from MicroScope were found only in population RT-I and RT-V, which match results from mapping using Geneious. The genes attributed to strain-specific regions found by PanSeq were all represented in the MicroScope output, with the exception of RT-II-2. This is a relatively small region (607 bp) and since MicroScope only looks at coding regions, it is possible this region comprises mostly non-coding DNA and was therefore not accounted for in the MicroScope output. The RGP Finder program in MicroScope looked for regions unique to a particular genome. The RGP regions found for each representative strain were mostly associated with novel regions found using PanSeq (Figure S2, Table 3), though some were not.

### 3.2. Average Nucleotide Identity and Digital DNA-DNA Hybridization

DNA-DNA hybridization (DDH) has been considered the gold standard for species delineation at the genomic level for the past 50 years, as it was the only method to offer a numerical and relatively stable species boundary [149]. Traditional DDH suffers from the fact that the method is laborious and requires specially trained personnel, who are only available in select laboratories. Recent advancements in next-generation sequencing (NGS) and bioinformatics have caused scientists to push for easier and more current methods. ANI has been suggested as the best alternative for a gold standard [149,150], as it can provide values that correlate to DDH values, with ~95%–96% ANI corresponding to the 70% DDH value for species delineation [67,149]. dDDH has also been suggested as a replacement for traditional DDH [82,83,84]. dDDH has an advantage over ANI in that the values are on the same scale as traditional DDH values, making comparison between digital and wet-lab results simple. Additionally, dDDH values calculated with GGDC have a higher correlation with traditional DDH. In this section, ANI and dDDH results were obtained from and compared using several independent programs. 

**Figure 2:**
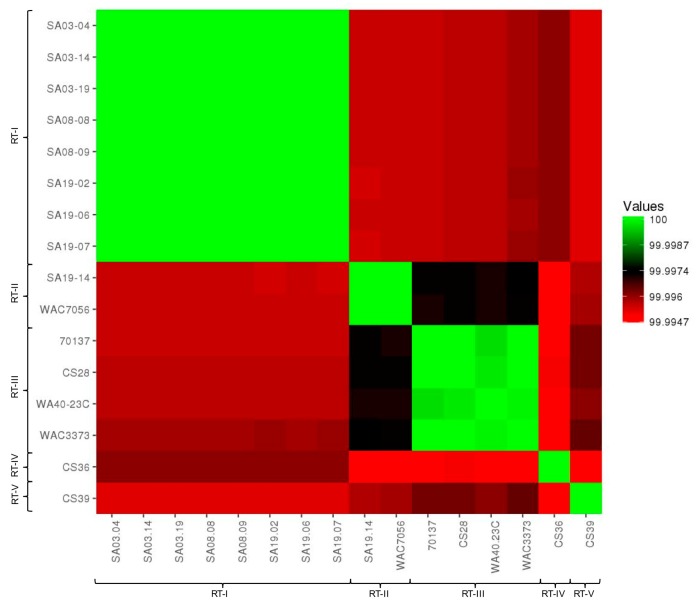
Heatmap of average nucleotide identities between *R. toxicus* strains used in this study. Average nucleotide identity (ANI) values calculated by the ANI calculator are color-coded according to the provided scale bar.

**Figure 3:**
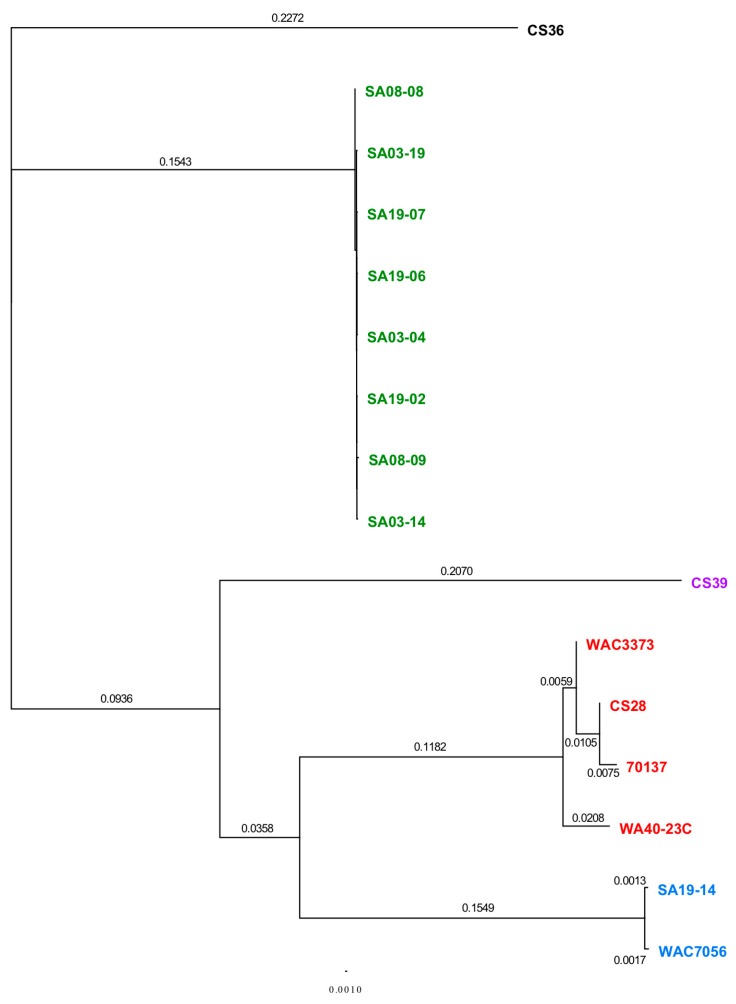
Phylogenetic tree based average nucleotide identity distances between *R. toxicus* strains used in this study. The ANI calculator was used to calculate ANI distances. The tree data were input into Geneious for phylogenetic tree generation. Strains within the phylogenetic tree are color-coded based on the genetic population. Green: RT-I; blue: RT-II; red: RT-III; black: RT-IV; purple: RT-V.

ANI calculator: ANI values were compared between all strain genomes tested using the ANI calculator (Table S15). ANI values produced by the ANI calculator were over 99.99% for all strain comparisons, which were higher than those produce by other analyses; higher ANI values from the ANI calculator were observed previously [150]. Nonetheless, ANI values were converted into a visual heat map for comparison (Figure 2). All strains grouped together with those of the same population type, reinforcing the population designations. Based on ANI values, RT-II and RT-III appear most closely related, as does RT-I and RT-IV. RT-V appears most closely related to RT-III and then RT-II; RT-V was most distantly related to RT-IV. This data is highly similar to that from pan-genome analyses. The ANI calculator data output includes a phylogenetic tree, which was imported into Geneious for tree generation (Figure 3). The phylogenetic tree produced from ANI distances shows the same trend; RT-II and RT-III are most closely related, with RT-V being the next closely related, while RT-I and RT-IV are most closely related. The web-interface for the ANI calculator also produces phylogenetic trees based on the NJ (Figure S5A) [112], BIONJ (Figure S5B) [151], and Unweighted Pair Group Method with Arithmetic Mean (UPGMA) (Figure S5C) [152] methods. The UPGMA tree is in complete agreement with Figure 3 and the ANI and pan-genome data with regards to population relatedness. The NJ and BIONJ trees have identical topologies that differ from the UPGMA tree; however, the relationships inferred from these trees are the same as the UPGMA tree, although lineages cannot be elucidated.

JSpecies: ANI values were compared between all strain genomes tested using JSpecies (Tables S16–S18). ANIb data (Table S16) suggests that RT-II and RT-III are most closely related among the five populations. RT-V was more closely related to RT-II and RT-III than to RT-I, and most distantly related to RT-IV. These results corroborate those found thus far. However, results suggest that RT-I is more closely related to RT-II and RT-III than RT-IV, though results for RT-IV show that it is still most closely related to RT-I. ANIm values (Table S17) corroborated the relationships between populations established by the ANI calculator and the pan-genome analysis. Tetranucleotide signatures were compared (Table S18) and results suggest that RT-II and RT-III are most closely related, with RT-V being more closely related to these populations and least related to RT-V. In contrast to other results, Tetra analysis suggests that RT-I is most closely related to RT-II; RT-IV also appears to be most closely related to RT-II. The only clear conclusions that could be drawn were that RT-II and RT-III were the most closely related and RT-IV and RT-V were the most distantly related. ANIb, ANIm and Tetra values were converted into visual heat maps for comparison (Figure S6A–C, respectively). All strains grouped together with those of the same population type, reinforcing the population designations.

EZGenome: ANI values were compared between all strain genomes tested using EZGenome (Table S19). The OrthoANIu values corroborated the relationships between populations established by the ANI calculator and the pan-genome analysis. OrthoANIu values were converted into a visual heat map for comparison (Figure S6D). All strains grouped together with those of the same population type, reinforcing the population designations.

Genome-to-genome distance calculator (GGDC): the GGDC was used to determine the similarity between strains/populations. BLAST+, the recommended default in GGDC, was used to calculate distance values, and then dDDH values were estimated according to three mathematical formulas (Tables S20–S25). dDDH values calculated based on Formula 1, 2 and 3 were converted into visual heat maps for comparison (Figure S7A–C, respectively). All strains grouped together with those of the same population type, reinforcing the population designations. Formula 1 and 2 produced dDDH values that suggested relationships congruent with each other, as well as previous analyses, suggesting RT-II and RT-III are most closely related, with RT-V being the next closely related, while RT-I and RT-IV are most closely related. In contrast, Formula 2 produced a relationship similar to those produced by ANIb, except that RT-IV was suggested to be more closely related to RT-I and RT-V was more similar to RT-III and then RT-I. It is interesting to note that GGDC recommends Formula 2 due to its independence of genome length, thus making it useful for analyzing incomplete draft genomes [82,83,84]. The GGDC suggests that for complete genomes, much higher DDH values for Formula 1 over Formula 2 would indicate that the strains differ less in gene content and more so in the sequences of their gene content [82,83,84]. GGDC suggests that Formula 2 will provide more resolution for strains that only differ by a plasmid [82,83,84]. These recommendations are confusing in that analyses of the *R. toxicus* genomes shows sequence differences, along with differences in gene content, yet the values for Formula 1 were larger than Formula 2, appearing to contradict statements made by GGDC. MUMmer was also used to determine distances and estimate dDDH values (Tables S26–S27); however, results were very unusual, as strains within the same populations were suggested to be least related. For example, the two RT-II strains have a dDDH value of 88.7 between them, while RT-II strains have dDDH values over 99% when compared to strains from all other populations. It is suggested to use the recommended BLAST+ analysis in GGDC to avoid erroneous results. GGDC also calculated differences in G+C content (Table S28); values were converted into a visual heat map for comparison (Figure S7D). Differences in G+C did not provide a conclusive resolution between populations. 

All analyses, except for GGDC MUMmer and G+C, agreed that RT-II and RT-III are most closely related, and RT-IV and RT-V are most distant. Varying relationships between other populations were observed depending on the program used; differences were observed between programs, as each program used slightly different algorithms for analysis, as well as between different formulas for calculating values within the same program. Another study observed these differences and suggests the use of multiple approaches for confirmation of taxonomic affiliations [150], which is the sentiment shared with this manuscript. It is believed that ANI is a robust measure of genetic and evolutionary distance; one reason being that it is not affected by HGT [67,153]. dDDH values are computed in a similar manner to ANI, therefore it can be assumed that dDDH may not account for HGT as well. Knowing now that HGT occurs frequently, it seems irresponsible to not account for it when analyzing the differences between two strains/populations; granted, current tools are limited in their ability to account for HGT events.

The fact that different programs gave different results and these calculations may not account for HGT led to further investigation into how these programs calculate ANI/dDDG values, in an attempt to understand how to interpret the data. Aside from reading the methods attributed to these programs, an experiment was performed to assess if and how each program accounts for HGT (Table S29). Strain SA03-04 was tested against itself as a control, and then the CRISPR-Cas region was deleted and compared with the unedited SA03-04 genome. The experiment was repeated with the CRISPR-Cas and tunicamycin gene cluster removed to test a larger deletion that occurs in two separate regions of the genome. As expected, all programs resulted in 100% identity values for the control experiment. ANI values from the ANI calculator were 100% for both experiments using the one-way ANI1 and two-way ANI. The one-way ANI2 produced an ANI value of 99.99% relative to the 100% control. There was a trend of decreasing fragment number analyzed as the amount of sequence removed from SA03-04 increased. When looking at the web-interface, the ANI value was ~100% when removing CRISPR-Cas alone or both the CRISPR-Cas and tunicamycin cluster. Strangely, removing both segments resulted in a slightly higher ANI percentage (99.9964184516498% vs 99.9963614798399%). The NJ, BIONJ and UPGMA trees grouped the modified SA03-04 genomes with the RT-I population, but the alterations were reflected in a slight shift in location relative to the others within the RT-I clade (Figure S8). ANIb and ANIm analyses from JSpecies showed a corresponding decrease in the percent genome assessed relative to the control as the amount of sequence removed increased; however, ANIb and ANIm values were 100% for both experiments. The Tetra values showed a corresponding decrease relative to the control, appearing to reflect the deletion made in SA03-04. OrthoANIu analysis from EZGenome was able to show a change relative to the control, reflecting the change in the analyzed genome; however, more of the genome was assed when both the CRISPR-Cas and tunicamycin were removed relative to when only the CRISPR-Cas system was removed. GGDC BLAST+ analysis showed no change relative to the control for the recommended Formula 2, thus showing that it does now account for HGT. Both Formulas 1 and 3 showed an increase in distance values and decrease in dDDH values relative to the controls that corresponded with increase deletion size, with both values being almost identical. The same trend was observed for BLAST+ and MUMmer percent G+C differences. GGDC MUMmer dDDH values reflected changes relative to the control when sections of the genome were removed; however, while the distance values increase with the corresponding increases in deletion, the dDDH values remained the same at 99.3%. These experiments demonstrate different programs may or may not account for genetic differences such as HGT within the system of interest; the extent to which these differences are represented is unknown. This again highlights the fact that multiple analyses should be performed in order to ensure accurate statements are made regarding the relatedness of organisms being studied. 

### 3.3. Phylogenetic Analyses Using Single Gene, Multi-Locus and Whole-Genome Sequence

**Figure 4:**
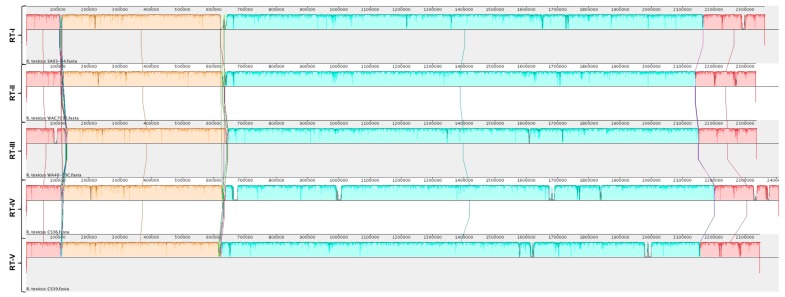
Genome alignment of strains representing *R. toxicus* populations RT-I, RT-II, RT-III, RT-IV and RT-V. Genome sequence alignments of representative strains of *R. toxicus* populations RT-I, RT-II, RT-III, RT-IV and RT-V were made in MAUVE using the progressive Mauve algorithm.

Phylogenetic analyses were used to determine the relationship among the *R. toxicus* strains used in this study. Complete genome sequences were aligned using MAUVE (Figure S9A). Strain 70137 (RT-III) appeared to have an inversion not present in any other strain; Sechler et al. [19] also showed this phenomenon. Simple sequence repeat (SSR) analysis of *R. toxicus* strain 70137 showed a drastic difference in repeat length when comparing a microsatellite identified within the sequence deposited in GenBank to its PCR verification results. Strain 70137 was isolated around the same time as CS28 and WAC3373, which have similar alignments. Being the only strain to possess this inversion, along with the SSR discrepancy, suggests that this anomaly is attributed to inaccurate sequence assembly. Therefore, a new alignment was performed in MAUVE that did not include strain 70137 (Figure S9B). No major inversions or rearrangements were observed for any *R. toxicus* strain tested, with the exception of strain 70137, which is consistent with results from Sechler et al. [19]. Strains of RT-I, RT-II and RT-III showed a high degree of similarity to others included in their respective populations; therefore, a MAUVE alignment was created using the strains representative of populations RT-I, RT-II, RT-III, RT-IV and RT-V (Figure 4).

**Figure 5:**
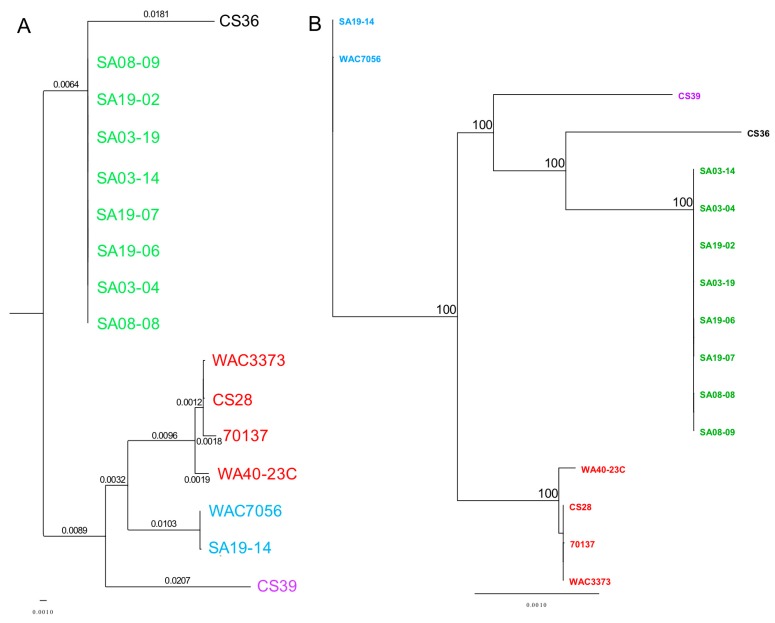
Whole-genome phylogenetic analysis of *R. toxicus* strains tested in this study. (**A**) Genome sequences were aligned with MAUVE and the tree data were input into Geneious for tree generation. (**B**) Genome sequences were analyzed using REALPHY. The REALPHY alignment data were input into Geneious for tree generation. Neighbor-joining (NJ) trees were calculated using the Jukes-Cantor method to compute evolutionary distances. Confidence intervals were assessed using the bootstrap method with 1000 replications. Strains within the phylogenetic tree are color-coded based on the genetic population. Green: RT-I; blue: RT-II; red: RT-III; black: RT-IV; purple: RT-V.

The MAUVE guide tree output and REALPHY alignment data were input into Geneious for phylogenetic tree generation (Figure 5). All strains grouped together with those of the same population type, reinforcing the population designations. Phylogenetic analysis by MUAVE shows that RT-II and RT-III are most closely related, while RT-V being next closely related (Figure 5A). MAUVE analysis also suggests that RT-I and RT-IV are most closely related (Figure 5A). These results agree with the majority of results presented thus far. REALPHY analysis also suggests that RT-I and RT-IV are most closely related; however, it also suggests that RT-V is the next closely related population (Figure 5B). MAUVE and REALPHY, which are both supposed to infer phylogenies based on whole-genome sequence, provide completely different topologies that suggest different relationships among populations. The phylogenetic tree produced by REALYPHY, as opposed to being created in Geneious using the REALPHY alignment file, has a similar topology to Figure 5B (Figure S10). The discrepancy between these two programs was most likely due to the fact that REALPHY looks at single nucleotide polymorphisms within the core; therefore, is unable to account for segments of the genome that do not match, such as those from HGT. This was tested in the same manner as the ANI/dDDH. REALPHY does not account for HGT, as SA03-04 with the CRISPR-Cas system removed, or both the CRISPR-Cas and tunicamycin gene cluster removed, grouped perfectly with the unedited SA03-04 strain within the phylogenetic tree. In contrast, MAUVE analysis reflected the deletion of the CRISPR-Cas system (Figure S11A) and both the CRISPR-Cas and tunicamycin gene cluster (Figure S11B), showing a corresponding increase in phylogenetic distance as more sequence was deleted.

MAUVE and REALPHY analysis showed strains CS28 and WAC3773 group close together with negligible distance within the clade; while 70137 groups with these two strains, it is separated from these two strains within the same clade by some phylogenetic distance. This was also observed from phylogenetic analyses using the ANI calculator (Figure S5C). These observed differences agree with the notion that the genome sequence of strain 70137 is inaccurate. Strain WA40-23C groups together with the other RT-III strains, but groups out separately in all phylogenetic analyses, including those from the ANI calculator. This is most likely due to the fact that this strain was isolated many years after the others, providing ample time to accumulate mutations that make its genome sequence different enough to be observed by these analyses. Similar findings were observed when analyzing the CRISPR spacer.

**Figure 6:**
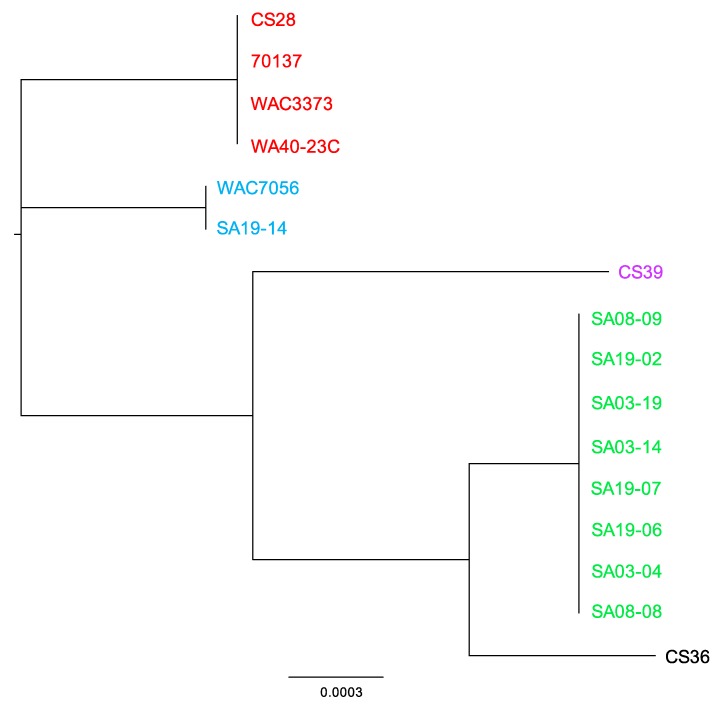
Phylogenetic analysis using PhyloSift. PhyloSift was used to analyze *R. toxicus* genomes, based on default parameters (maximum-likelihood analysis). The output tree file was input into FigTree v1.4.3 (tree.bio.ed.ac.uk/software/figtree/) to produce the phylogenetic tree. Strains within the phylogenetic tree are color-coded based on the genetic population. Green: RT-I; blue: RT-II; red: RT-III; black: RT-IV; purple: RT-V.

Complete *R. toxicus* genome sequences were also analyzed using PhyloSift, under default conditions. The output tree file was input into FigTree v1.4.3 (tree.bio.ed.ac.uk/software/figtree/) to display the Maximum-likelihood tree (Figure 6). PhyloSift analysis grouped all strains with their respective populations, which is congruent with all other analyses mentioned previously. Based on the topology of the phylogenetic tree produced by PhyloSift, RT-I and RT-IV appear most closely related, followed by RT-V, and then RT-II and RT-III. Unlike some of the analyses mentioned previously, there were no intra-population differences observed, particularly within RT-III. The inability of PhyloSift to show differences among RT-III, particularly regarding recent strain WA40-23C, can most likely be attributed to the conserved nature of the gene families used during analyses. For prokaryotic analysis, PhyloSift uses a reference database of 37 gene families that were found to be single copy, almost without exception, and essentially universal [154]. These genes comprise mostly ribosomal proteins, translation elongation and initiation factors (EF-2 and IF-2), a metalloendopeptidase, *ffh* signal recognition particle, phenylalanyl-tRNA synthetase alpha and beta subunits, tRNA pseudouridine synthase B, porphobilinogen deaminase, phosphoribosylformylglycinamidine cyclo-ligase and ribonuclease HII. PhyloSift databases also include 16S and 18S ribosomal RNA genes, as well as mitochondrial, Eukaryote-specific and viral gene families [115]. As PhyloSift only analyzes genes contained within its database and which are common to all strains, it is likely that this platform cannot account for horizontal gene transfer events. This was tested in the same manner as the ANI/dDDH, MAUVE and REALPHY, by rerunning analyses with an SA03-04 sequence devoid of the CRISPR-Cas system or both the CRISPR-Cas system and tunicamycin gene cluster. Both analyses showed the altered sequence grouped perfectly with the unaltered SA03-04 sequence, suggesting the large deletions were not accounted for during analysis if not occurring among the preselected genes.

**Figure 7:**
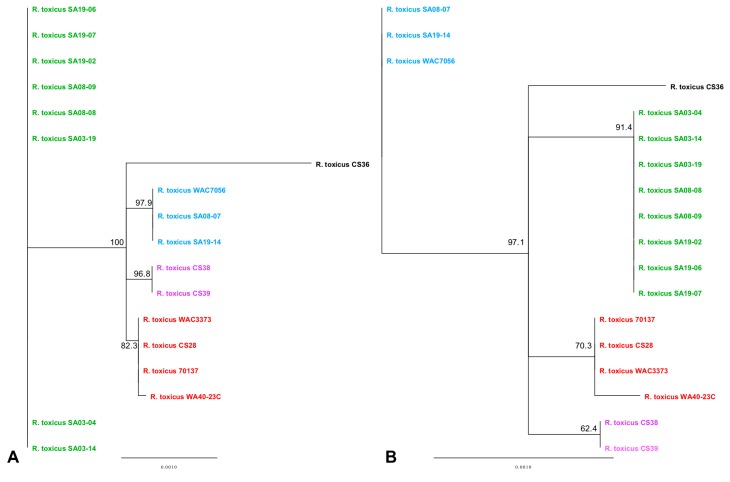
Multi-locus sequence analysis of *R. toxicus* isolates used in this study. Neighbor-joining trees were based on concatenated (**A**) DNA and (**B**) amino acid sequences of six genes (*atpD*, *dnaK*, *gyrB*, *ppK*, *recA* and *rpoB*) from 18 *R. toxicus* isolates and calculated using the Jukes-Cantor method to compute evolutionary distances. Confidence intervals were assessed using the bootstrap method with 1000 replications. Strains within the phylogenetic tree are color-coded based on the genetic population. Green: RT-I; blue: RT-II; red: RT-III; black: RT-IV; purple: RT-V.

Multi-locus sequence analysis has been suggested as the new gold standard for species delineation [155,156]; however, one of the major drawbacks is gene selection bias [149]. Arif et al. [35] performed MLSA using the partial gene sequences of the chromosome partition protein SMC, CRISPR-associated protein *cse4*, cysteine desulfurase, *secA* ATPase, tRNA dihydrouridine synthase, and vancomycin A-type resistance protein *vanA*. These genes were chosen based on their discriminative power, variety of cellular function and spatial coverage of the entire genome [35]. As with many studies, attempts are made to minimize gene bias by selecting genes of various function and spatial distribution throughout the genome. PhyloSift removes selection bias by analyzing a large number of preselected genes, regardless of the system used. In an attempt to minimize gene selection bias, MLSA was performed using genes selected for another system, *Clavibacter michiganenis* [157,158]; *Rathayibacter* spp. once grouped within the *Clavibacter* genus [1,8,159]. MLSA analysis of six full gene sequences was able to reproduce the population grouping for RT-I, RT-II, RT-III, RT-IV and RT-V (Figure 7); results revealed different topology from PhyloSift and previous MLSA results [35,36] (Figure S12), thus suggesting different relationships between populations. Results are based on only 46 unique SNPs (Figure 7A), which corresponds to 13 unique amino acid substitutions (Figure 7B).

Each gene included in the MLSA was also analyzed individually (Figure S13). Analysis of *atpD* (1476 bp), *dnaK* (1863 bp), *gyrB* (2061 bp), *ppK* (2199 bp), *recA* (1068 bp) and *rpoB* (3492 bp) was based on 2, 9, 18, 9, 2 and 6 unique SNPs, respectively (Figure S13A–F), which corresponded to 0, 1, 3, 1, 2 and 6 amino acid substitutions, respectively. Interestingly, Arif et al. [35] screened *dnaK* and *rpoB* for their ability to differentiate populations and found no differences in nucleotide sequence; results were based on analysis of a 951 and 871 bp gene fragment, respectively. The *dnaK* gene contained only one unique SNP in RT-I and eight unique SNPs in RT-IV; therefore, not much resolution was provided (Figure S13B). In contrast, the *rpoB* gene alone could separate all strains into their respective populations (Figure S13F); however, distances separating the clades is based on only six total unique SNPs, which corresponded to six total unique amino acid substitutions.

Production of the tunicamycin toxin is the main reason *R. toxicus* has received attention in the past. How this organism utilizes this toxin remains unknown. It is speculated that the toxin is used to compete with the nematode or other microorganisms. As different populations may be exposed to different vectors, plant hosts and other competing microorganisms depending on geographic location, the tunicamycin gene cluster was analyzed for potential differences that may reflect this notion (Figure S14). The full tunicamycin gene cluster was analyzed (Figure S14A) and grouped strains of *R. toxicus* into their respective populations; however, RT-II and RT-III strains grouped together in a single clade. Analysis also revealed some sequence length differences attributed to a poly-G repeat portion that could be a sequencing artifact. Therefore, individual tunicamycin gene coding sequences were extracted and analyzed individually; the order of tunicamycin genes were as described previously (*tunC-A-B-K-G-H-L-D-E-I-J-F*) [19]. Individual tunicamycin genes possess very few SNPs, with most occurring in RT-IV. Individual analyses did not provide resolution of *R. toxicus* populations; therefore, *tun* gene coding sequences were concatenated for reanalysis (Figure S14B). Concatenated sequences were also translated into aminoacid sequences and analyzed (Figure S14C). Nucleotide and amino acid analyses of concatenated *tun* gene coding sequences showed identical groupings of strains, which was also congruent with those produced when analyzing the full tunicamycin gene cluster; topologies varied slightly.

Tunicamycin production was thought to be attributed to the presence of a bacteriophage [160]; however, a recent study identified toxin-producing isolates without the bacteriophage [161]. To date, the trigger for the production of tunicamycin by the bacterium remains unknown, though toxin seems to appear as seeds are senescing [21]. Secondary metabolites tend to be produced during times of stress. Whether the stress is attributed to phage infection or senescence during the natural lifecycle remains unclear. Trehalose is a non-reducing disaccharide that can accumulate in bacteria during stress, such as osmotic stress experienced during seed senescence, to enable survival [162]. Bacteria possess five trehalose biosynthetic pathways [162]; *R. toxicus* has two trehalose biosynthetic pathways (Figure S15). Specific plant hosts may senesce differently, which could be reflected in how *R. toxicus* populations deal with this stress through their trehalose pathways; therefore, each pathway was analyzed (Figure S16). The *tps* has five total SNPs, one in RT-II/IV, two in RT-III/IV and two only in RT-IV, corresponding to only one amino acid substitution in RT-IV. *tps* analysis grouped each strain with their respective population; however, RT-II and RT-V were grouped into a single clade (Figure S16A). The *tpp* has 17 total SNPs, one in RT-V, two in RT-II/III and 14 in RT-IV, corresponding to 7 amino acid substitutions in RT-IV and only one amino acid substitution in RT-V. *tpp* analysis also grouped each strain with their respective population; however, RT-II and RT-III were now grouped into a single clade (Figure S16B). Interestingly, when *tps* and *tpp* were concatenated, strain grouped with their respective populations (Figure S16C), providing further validation of the five genetically distinct populations; however, phylogenetic topology differed from larger-scale analyses.

**Figure 8:**
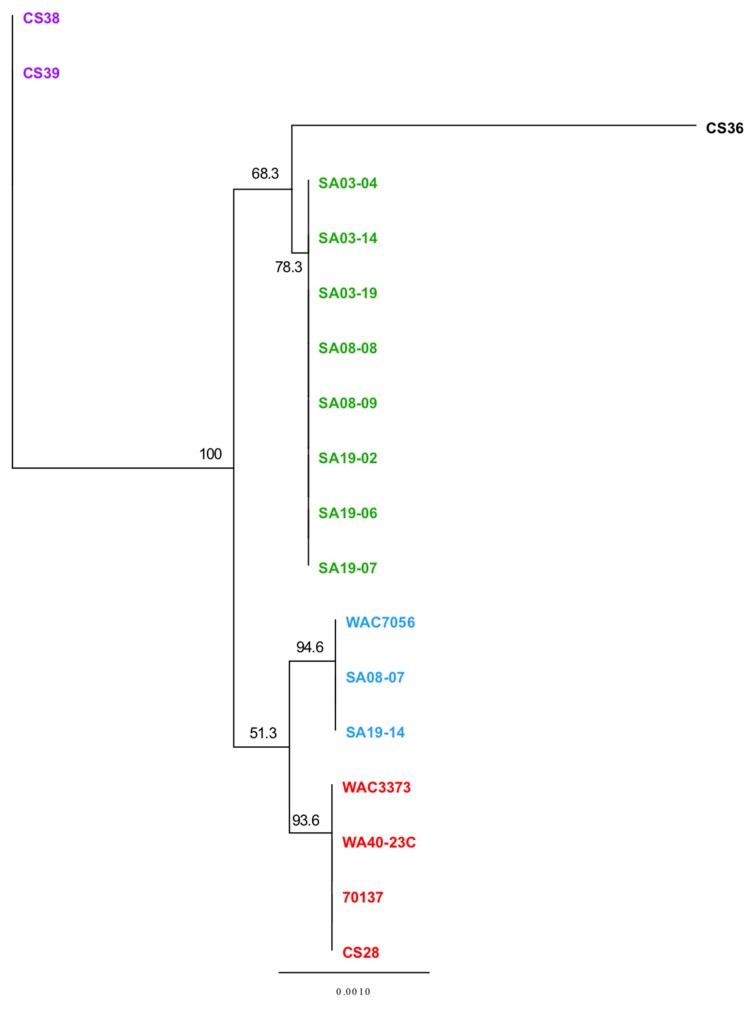
Phylogenetic analysis of the *R. toxicus* trehalose biosynthetic pathway genes. Neighbor-joining trees were based on concatenated DNA sequences of four tunicamycin biosynthesis genes (*tps*, *tpp*, *treY* and *treZ*) from 18 *R. toxicus* isolates and calculated using the Jukes-Cantor method to compute evolutionary distances. Confidence intervals were assessed using the bootstrap method with 1000 replications. Strains within the phylogenetic tree are color-coded based on genetic population. Green: RT-I; blue: RT-II; red: RT-III; black: RT-IV; purple: RT-V.

The *treY* has eight total SNPs, two in RT-I/IV and six in RT-V, corresponding to six amino acid substitutions in only RT-V. The *treY* analysis grouped RT-II and RT-III together, as well as RT-I and RT-IV (Figure S16D). *treZ* has three total SNPs, one in RT-II and two in RT-V, corresponding to only one amino acid substitution in RT-II. *treZ* analysis was less informative, with RT-I, RT-III and RT-IV grouping together (Figure S16E). Concatenating *treY* and *treZ* grouped each strain with their respective population; however, RT-I and RT-IV were now grouped into a single clade (Figure S16F). Individual analyses of amino acid sequences did not provide resolution of *R. toxicus* populations. The analysis of individual genes from each pathway was based on only a few SNPs; therefore, all four genes were concatenated and reanalyzed (Figure 8). All tested strains grouped with their respective populations and the topology seemed to mostly agree with those provided by larger-scale analyses, with RT-I and RT-IV being most closely related, as is RT-II and RT-III. Again, due to the few numbers of SNPs involved in this analysis, concatenated amino acid sequences of the four genes produced conflicting results; strains grouped with their respective populations, but RT-I and RT-III grouped together (Figure S17). Again, this reinforces the point that phylogenetic relationships can differ depending on the type and number of genes used in a study.

Exopolysaccharide (EPS) interacts with the environment (vector, host, etc.) and may play a role in protection during dehydration as seeds senesce. The exopolysaccharide production protein was analyzed and revealed five total SNPs, three in RT-I, one in RT-I/IV and one in RT-II; each SNP corresponded to an amino acid substitution. Strains grouped with their respective populations (Figure S18A) and the inferred phylogenies seemed to mostly agree with those provided by larger-scale analyses, with RT-I and RT-IV being most closely related; however, RT-III grouped with RT-V. Amino acid analysis produced identical groupings, but the topology was reversed.

Vancomycin resistance genes (*vanHAX*) are possessed by all *R. toxicus* isolates, though not present in *R. festucae*, *R. iranicus*, *R. rathayi*, and *R. tritici*; presence of vancomycin resistance genes in the remaining *Rathayibacter* spp. is unknown. Each vancomycin gene was assessed individually. The full vancomycin cassette was assessed and revealed only five total SNPs, corresponding to only a single amino acid change in RT-I/IV in the *vanX* gene. Phylogenetic analysis of individual genes was uninformative. The full vancomycin cluster grouped strains with their respective populations; however, RT-II and RT-V grouped together into a single clade (Figure S18B). It appears that selective pressure is not only strong enough for *R. toxicus* to retain these genes, but keep the gene sequenced conserved. Out of curiosity, EPS and vancomycin genes were concatenated and analyzed; all strains grouped with their respective populations and all populations formed unique clades. It should be noted that the vancomycin gene cassette (*vanHAX*) was inverted in only strain 70137; this is consistent with other analyses that found inversions and other errors in the published genome sequence.

All the genes tested individually thus far, including those used in MLSA, were analyzed by BLAST to determine where the genes may have been inherited from and/or what organisms closely share these gene sequences (Table S30). The CRISPR-Cas cassette was acquired by HGT; therefore, Cas genes were also included in this analysis (Table S30). Under parameters to identify highly similar matches, majority of the genes analyzed matched only to the two complete *R. toxicus* genomes (70137 and WAC3373) deposited on GenBank, with the exceptions being *vanA*, *tps*, *treY* and *treZ*, as well as *tpp* in RT-IV. *vanA* matched to *Sacchrothrix espanaensis* DSM 44299 with an identity and query coverage of 70% and 90%, respectively, while the TPP of RT-IV matched to *R. tritici* NCPPB 1953 with an identity and query coverage 77% and 91%, respectively. Interestingly, the other trehalose genes also matched with *R. iranicus*, with identities and query coverages of 74%–78% and 88%–97%, respectively. The *cas* and *tun* genes did not yield other matches, knowing that they were acquired horizontally. This may indicate/reflect that these genes were acquired long ago, and that sufficient time has passed to allow for the accumulation of enough SNPs that sequences no longer resemble that of the original acquisition.

Variable results were obtained for all genes when analyzed under less stringent conditions using the “Somewhat similar sequences (blastn)” option within NCBI BLAST. ESP matched with *Agromyces aureus* AR33, *R. festucae* DSM 15932, *R. rathayi* DSM 7485, *R. iranicus* NCPPB 2253 and *R. tritici* NCPPB 1953, but with varying query coverage (17%–65%) and identities (68%–74%). The individual vancomycin genes yielded various matches from a variety of organisms (none of the other *Rathayibacter* spp.), with high query coverages (>70%) and ~70% identity. Trehalose genes only matches with few different organisms, including *R. festucae*, *R. rathayi*, *R. iranicus*, *R. tritici*, *Clavibacter*, *Plantibacter* and *Leifsonia*, which were all part of the *Clavibacter* genus at one point in time [1,8,159,163,164], with high query coverages (~60%–98%) and high identities (~65%–79%). This suggests that trehalose genes belonged to an ancient ancestor. The *tun* and *cas* genes yielded interesting results. The *cas3* and *cse1-cse3* genes also showed no matches under less stringent parameters. The *cas5e* gene only produced a single match to *Actinomyces* with relatively low query coverage (53%) and identity (66%), while the remaining genes matched a few different organisms with varying levels of query coverage and identity; matches to *Nocardia* were common. Under relaxed BLAST parameters, no matches were found for *tunE*, *tunG*, *tunH* and *tunI*. The remaining tunicamycin genes matched to the genome of *R. iranicus* NCPPB 2253, with *tunA*, *tunB*, *tunC*, *tunD*, *tunF*, *tunJ*, *tunK* and *tunL* having varying query coverages (16%, 97%, 25%, 11%, 90%, 65%, 22% and 59%, respectively) and identities (67%, 72%, 68%, 68%, 63%, 68%, 82% and 65%, respectively). *tunB* also matched to *Streptomyces*, *Nocardia* and *Actinosynnema* with high query coverage (~97%) and identity (~64%). This was expected as it was shown previously that *R. toxicus* tunicamycin genes are most closely related to that of *Streptomyces chartreusis* [19]; lack of any match to *Streptomyces* for other *tun* genes was unexpected. Tunicamycin production by *R. iranicus* has not been confirmed, but it is suspected to be able to produce corynetoxin [21]. BLAST data may suggest this possibility; however, mapping *tun* genes to *R. iranicus* NCPPB 2253 was unsuccessful. Any potential tunicamycin gene sequence, length and arrangement within the genome of *R. iranicus* NCPPB 2253 appear to differ considerably when compared to that of *R. toxicus*, based on searching for functional annotations similar to those of *tun* genes.

The lack of matches to anything except the two *R. toxicus* genomes for all *cas* and *tun* genes under stringent BLAST conditions, along with the few or no significant matches under less stringent conditions, suggest that these genes, whether acquired vertically or horizontally, were present in *R. toxicus* for a significant amount of time to allow accumulation of enough mutations to make these sequences unique to this organism. If it is assumed horizontally transferred genes such as *cas* and *tun* were acquired at or just after the split of *R. toxicus* from other *Rathayibacter* spp., as all others lack these genes, these genes could have been acquired 18–33 million years ago; the difference in G+C content between *R. toxicus* and *R. festucae*, *R. iranicus*, *R. rathayi* and *R. tritici* is approximately 11%, 6%, 8% and 8%, respectively, and studies suggest that genome-wide changes in G+C content in prokaryotes occur at a rate of approximately 1% every 3 million years [165,166].

Manual annotation of the core-genome led to the discovery of genes with interesting functional annotations, prompting further investigation; these genes had functions involved with cell wall synthesis, secretion systems and toxins, cell cycle, antibiotic resistance and phage-related proteins. Sequences for the cyclic-di-GMP-binding biofilm dispersal mediator protein, cell division trigger factor and cell division inhibitor protein were 100% identical; other genes shared varying numbers of SNPs: phage-shock protein PspC, cell division inhibitor protein and zeta toxin contained a single SNP. These genes were found in the core-genome designated by MicroScope, but cyclic-di-GMP-binding biofilm dispersal mediator protein could not be mapped to strain 70137. The core-genome was assessed using representative strains of each *R. toxicus* population, so it is possible that 70137 does not have this gene or sequence variation prevents proper mapping of this gene to strain 70137 using Geneious. As the sequence for this gene was found to be 100% identical in the other 17 *R. toxicus* strains tested, it is likely that errors within the 70137 sequence are responsible; other errors found in the 70137 sequence were explained previously. Phylogenetic analysis of these genes was not informative.

The T3SS SseB-like protein sequence was very large (6783 nt) and contained many SNPs; the *sse* gene is a *Salmonella enterica* effector associated with assembly of an F-actin coat around intracellular bacteria. Phylogenetic analysis was able to group all strains into their respective populations, with RT-I being most closely related to RT-IV (Figure S19A). Additionally, the topology of the phylogenetic tree suggests that RT-IV and RT-V are the most distant (Figure S19A); results corroborate previous analyses. This gene may warrant further investigation. MLSA was performed on 13 of these genes, excluding the three genes with 100% identity and the T3SS SseB-like protein. Concatenated sequences (15,795 nt) were analyzed and resulting phylogenies grouped all strains with their respective populations; topology suggested different relationships among the populations. Analysis was performed again, now including the T3SS SseB-like protein (14 genes). Concatenated sequences (22,578 nt) were analyzed and resulting phylogenies grouped all strains with their respective populations (Figure S19B). The topology of the phylogenetic tree produced from MLSA of these 14 genes suggests the same relationships as gene family content analysis by MicroScope; RT-IV ↔ RT-I ↔ RT-II ↔ RT-III ↔ RT-V. Strain WA40-23C grouped within the RT-III clade but separate from all other strains within this clade. This observation can be attributed to the inclusion of the kojibiose/trehalose phophorylase gene. This gene contained nine total unique SNPs; WA40-23C had its own unique SNPs which caused it to group out separately from all other strains. 

The T3SS SseB-like protein sequence was very large (6783 nt) and contained many SNPs; the *sse* gene is a *Salmonella enterica* effector associated with assembly of an F-actin coat around intracellular bacteria. Phylogenetic analysis was able to group all strains into their respective populations, with RT-I being most closely related to RT-IV (Figure S19A). Additionally, the topology of the phylogenetic tree suggests that RT-IV and RT-V are the most distant (Figure S19A); results corroborate previous analyses. This gene may warrant further investigation. MLSA was performed on 13 of these genes, excluding the three genes with 100% identity and the T3SS SseB-like protein. Concatenated sequences (15,795 nt) were analyzed and resulting phylogenies grouped all strains with their respective populations; topology suggested different relationships among the populations. Analysis was performed again, now including the T3SS SseB-like protein (14 genes). Concatenated sequences (22,578 nt) were analyzed and resulting phylogenies grouped all strains with their respective populations (Figure S19B). The topology of the phylogenetic tree produced from MLSA of these 14 genes suggests the same relationships as gene family content analysis by MicroScope; RT-IV ↔ RT-I ↔ RT-II ↔ RT-III ↔ RT-V. Strain WA40-23C grouped within the RT-III clade but separate from all other strains within this clade. This observation can be attributed to the inclusion of the kojibiose/trehalose phophorylase gene. This gene contained nine total unique SNPs; WA40-23C had its own unique SNPs which caused it to group out separately from all other strains. 

## 4. Conclusions

*Rathayibacter toxicus* is aptly named and unique among *Rathayibacter* species, in that five populations contain a functional tunicamycin gene cluster, evident by the livestock toxicities reported at each of the geographic locations where the bacterium has been detected. All five *R. toxicus* populations also contain a CRISPR-Cas system and vancomycin resistance genes; the CRISPR-Cas system appears most similar to a type I-E CRISPR-Cas system (*Escherichia coli*-type) [19]. *Rathayibacter toxicus* is the only *Rathayibacter* spp. containing these gene clusters, suggesting their acquisition from an HGT event that occurred early in the speciation of *R. toxicus* away from other *Rathayibacter* spp.

The purpose for retention of the tunicamycin gene cluster by *R. toxicus* remains unknown. Researchers have been unable to recreate the lifecycle of this pathogen in vitro, which includes the bacterium, a nematode vector and a plant host. This limits the ability to determine the role of the toxin in the life history of the bacterium. *R. toxicus* tunicamycin genes were shown to be closely related to that of *Streptomyces chartreusis* [19]; *S. chartreusis* is an Actinobacteria that was isolated from soil in Africa. Although only confirmed in Australia, the fact that *R. toxicus*-like disease was reported in South Africa, along with the similarity of the tunicamycin cluster to that of *S. chartreusis*, may suggest *R. toxicus* originated in South Africa. Exactly when and how this may have occurred requires further investigation; this could have been caused by movement of animals or plant host species, such as *Ehrharta longiflora* [167], into Australia. Another uncharacterized species of *Rathayibacter*, *Rathayibacter* sp. EV, was isolated by Riley et al. [168] from *Ehrhata villosa* var. *villosa* in South Africa and was shown to be vectored by *Anguina woodi*, but not *Anguina funesta* or *Anguina tritici*. *Rathayibacter* sp. EV, along with *Rathayibacter iranicus*, are suggested to be able produce tunicamycin [21]. Perhaps *Rathayibacter* sp. EV represents a population similar to the ancestral population, or it diverged from the common ancestor on a separate evolutionary path from populations in Australia. It is crucial that future studies include isolates from South Africa to either confirm or deny these statements.

To date, five distinct populations of *R. toxicus* have been identified based on multiple analyses, including AFLP [31], PFGE [31], MLSA [35,36], SNP [28,30] and ISSR analysis [35]. This study represents the first large-scale genome-wide study of *R. toxicus* to include strains from populations RT-I and RT-V. Genomes were analyzed using a variety of methods and bioinformatics tools. In regard to the conclusions, the term “method” is used to describe the type of analysis used to query the genome, such as ANI, MLST or whole-genome. The term “tool” refers to the software program used to perform a given method of analysis, such as using JSpeciesWS for ANI calculation or MAUVE for whole-genome analysis. The term “algorithm” is used to describe the mathematical formulas inherent and built into a given tool. Algorithms can differ between tools used for a given method, such as differing between the ANI calculator and EZGenome even though both are used to calculate ANI; these can also differ for a given tool, such as ANIb or ANIm, which are based on BLAST+ or MUMmer for JSpeciesWS, respectively.

All large-scale analyses grouped strains into five distinct populations. For any given set of analyses, only subtle differences were usually observed between programs; however, these subtle differences altered inferred relationships among these populations. The use of different annotation programs resulted in varying gene counts, gene locations, and functional annotations. The use of different algorithms by various bioinformatic tools used to calculate ANI and dDDH produced varying relationships among populations.

Significant differences were obtained for HGT and prophage/phage remnant analyses depending on the analytical tool used; there was no instance where all programs agreed. Lack of concordance was even more apparent between programs used to identify prophage and phage remnants. Three bioinformatics tools identified prophage/phage remnants; however, there was no agreement between them, as they each identified different sequences of different sizes and at different locations within the genome. In addition, there was a lack of consistency among and within programs. In some instances, sequences were not identified in a given strain even though it was actually present. One specific example is strain SA03-14 when analyzed by AntiSMASH 4.0. The majority of the BGCs were not identified in this strain but were identified by mapping these BGCs from other strains to the SA03-14 genome in Geneious. This was also observed to varying degrees during prophage analyses. These observations, along with SSR analysis of strain 70137, emphasize the importance of verifying results by other means, such as PCR or mapping known sequences to a query genome, to ensure the presence or absence of a locus, as well as its sequence accuracy. Another observation to note was the differences in results produced by different versions of the same program; AntiSMASH 4.0 and AntiSMASH 4.2.0 produced different results, while PHASTER, a more advanced version of PHAST with a very similar phage identification pipeline, did not identify prophage sequence found by PHAST.

In this study, different methods and different versions of the same software programs occasionally produced different results, sometimes leading to different conclusions. These observations indicate a certain risk in using a single method for analysis, not only the analytical method itself but also the tools used for that analysis. Additionally, it is important to understand how these tools function (e.g., what is included in the analysis and what is not considered) and the purpose for which they were designed. Understanding the limitations of the analytical tools will allow informed conclusions to be drawn from a particular analysis. Phylogenetic analyses like PhyloSift, REALPHY and MLSA did not account for HGT events during analyses; scientists now agree that HGT events occur at rates far higher than believed previously. Additionally, it is important to know whether an analytical tool considers other aspects of the genome, such as inversions and rearrangements, as the orientation and organization of the same genes can differ among strains. Many analyses do not account for these genome characteristics: they are more focused on the presence/absence of genes and the sequence differences; thus, the true phylogeny of the organism may not be accurately reflected. It is also important to note that even though phylogenetic relationships can be established with these analyses, evolutionary lineages cannot always be directly inferred; strain data and historical data need to be assessed together with the genetic data to infer evolutionary trajectories. For example, knowing the ages of the isolates, the location of isolates, and the historical movement of host material, can assist in putting the genetic data into perspective.

Many studies rely highly on MLSA data. Results obtained in this study demonstrate that depending on the type and number of genes used, phylogenetic relationships can differ. It seems apparent that including more genes in an MLSA would increase the resolution and provide results comparable to the true nature of the organism being studied; however, there appear to be no standards for MLSA. Additionally, results may vary depending on the PCR primers used to sequence the same gene, as different primers can target completely different regions within the same gene. Arif et al. [35] analyzed partial *dnaK* and *rpoB* gene sequences that were amplified using specific primers and saw no difference in sequence among the tested strains; however, this study showed apparent differences, particularly in the *rpoB* gene, when the full gene sequence was used.

Cumulative data from this study reinforce the existence of these five genetically distinct populations and suggest RT-II and RT-III, and then RT-V, are more closely related to each other than other populations, while RT-I and RT-IV are more closely related to each other than other populations. Cumulative data also suggest that RT-IV and RT-V are the most unrelated among the five populations. The idea that RT-III evolved from RT-II and reached Western Australia from South Australia through the movement of ryegrass is consistent with historical and genetic data; this conclusion was also reached by Davis II et al. [28,30]. Additionally, Davis II et al. [28,30] suggests that *R. toxicus* tends toward genome reductions. If true, the fact that population RT-IV has the largest genomes compared to other populations, followed by RT-V, would support the idea of *R. toxicus* populations evolving in Australia from RT-IV, as well as populations RT-II and RT-III arising from RT-V. A comprehensive analysis of CRISPR-Cas system also suggests this pattern of evolution. Although only two strains each were available for populations RT-IV and RT-V, every analysis performed supported their identification as distinct populations.

Apart from the co-existence of RT-I and RT-II on the Yorke Peninsula in South Australia, the remaining *R. toxicus* populations are geographically isolated; RT-III in Western Australia, RT-IV in New South Wales, and RT-V in a small area of southeast South Australia. This geographic isolation may have contributed to the genetic differences among populations. Future research will involve attempts to collect additional isolates belonging to populations RT-IV and RT-V, as well as regular sampling over time to track evolutionary changes.

## Figures and Tables

**Table 1:** *Rathayibacter toxicus* strains used in this study.

ID	Other ID	Group ^a^	Host ^b^	Year	Location ^c^	Collector	GenBank Accession
SA03-04		RT-I	ARG	2014	Corny Point, SA	M. Arif/J. Stack	CP037987
SA03-14		RT-I	ARG	2014	Corny Point, SA	M. Arif/J. Stack	CP037986
SA03-19		RT-I	ARG	2014	Corny Point, SA	M. Arif/J. Stack	CP037985
SA08-08		RT-I	ARG	2014	Lake Sunday, SA	M. Arif/J. Stack	CP037984
SA08-09		RT-I	ARG	2014	Lake Sunday, SA	M. Arif/J. Stack	CP037983
SA19-02		RT-I	ARG	2013	Yorketown, SA	M. Arif/J. Stack	CP037982
SA19-06		RT-I	ARG	2013	Yorketown, SA	M. Arif/J. Stack	CP037981
SA19-07		RT-I	ARG	2013	Yorketown, SA	M. Arif/J. Stack	CP037980
SA08-07		RT-II	ARG	2013	Lake Sunday, SA	M. Arif/J. Stack	N/A
SA19-14		RT-II	ARG	2013	Yorketown, SA	M. Arif/J. Stack	CP037979
WAC7056 (type strain)	SAC7056, CS14, ICMP 9525, JCM9669, NCPPB 3552, D84127, FH49, FH137	RT-II	ARG	1983	Murray Bridge, SA	I.T. Riley	CP037977
70137	WSM447, CS30, FH145, WAC3396	RT-III	Oat	1980	Gnowangerup, WA	D. Chatel	CP010848
CS28	ICMP 6307, FH138	RT-III	ARG	1978	WA	I.T. Riley	CP037990
WA40-23C		RT-III	ARG	2015	WA	M. Arif/J. Stack	CP037978
WAC3373	WSM194	RT-III	PG	1978	Gnowangerup, WA	D. Chatel	CP013292
CS36	SE-1	RT-IV	PBG	1990	Gongolgon, NSW	A. McKay	CP037989
CS38	NSW-11	RT-IV	ABG	1990	Lucindale, SA	A. McKay	N/A
CS39	NSW-16	RT-V	ABG	1990	Lucindale, SA	A. McKay	CP037988

^a^ Groups based on population designations from Arif et al. 2016 [35]; RT-IV and RT-V designations from Yasuhara–Bell and Stack 2019 [36]. ^b^ PBG—Pacific bent grass (*Agrostis avenacea*); ABG—annual beard grass (*Polypogon monspeliensis*); ARG—annual ryegrass (*Lolium rigidum*); PG—paradoxa grass (*Phalaris paradoxa*); Oat (*Avena sativa*). ^c^ NSW—New South Wales; SA—South Australia; WA—Western Australia.

**Table 2:** Genome analysis of representative strains of *R. toxicus* populations by MicroScope.

Strain	Population	Pan CDS	Core CDS	Core CDS %	Variable CDS	Variable CDS %	Strain Specific CDS	Strain Specific CDS %
SA03-04	RT-I	2666	2157	80.908	509	19.092	111	4.164
WAC7056	RT-II	2605	2160	82.917	445	17.083	67	2.572
WA40-23C	RT-III	2592	2153	83.063	439	16.937	82	3.164
CS36	RT-IV	2694	2182	80.995	512	19.005	154	5.716
CS39	RT-V	2620	2160	82.443	460	17.557	115	4.389

**Table 3:** Unique regions determined by PanSeq for each population of *R. toxicus*.

Region ^a^	RT-I	RT-II	RT-III	RT-IV	RT-V	Size (bp)	Notes
**RT-I-1**	x					799	In ASC-2; ASC-2 longer in RT-V than all others but missing RT-I-1
**RT-I-2**	x					945	
**RT-I-3**	x					749	In ASC-4
**RT-I-4**	x					1661	In ASC-6, SA03-04 RGP-1; CS36 RGP-1
**RT-I-5**	x			x		3604	In ASC-6, SA03-04 RGP-1; CS36 RGP-1
**RT-I-6**	x			x		19,556	Partly in ASC-13; SA03-04 RGP-2; CS36 RGP-2; LAMP primers designed here [36]
**RT-I-7**	x					1791	Between TreY-TreZ and ASC-14; LAMP primers designed here [36]
**RT-I-8**	x			x	x	1679	In ASC-16; In RT-IV, RT-I-8 partially at end of ASC-16 and also after ASC-20
**RT-I-9**	x					1023	
**RT-I-10**	x					940	
**RT-II-1**		x				604	LAMP primers designed here [36]
**RT-II-2**		x				607	In ASC-4
**RT-II-3**		x		x	x	4863	In ASC-15; LAMP primers designed here [36]
**RT-II-4**		x		x	x	4449	In ASC-15
**RT-II-5**		x			x	1522	Slight overlap within WAC7056 RGP-3 and CS39 RGP-2
**RT-II-6**		x			x	1371	
**RT-III-1**			x			8944	In ASC-2; WA40-23C RGP-1
**RT-III-2**			x			1329	In ASC-2; WA40-23C RGP-1; LAMP primers designed here [36]
**RT-III-3**			x	x	x	1908	
**RT-III-4**			x	x	x	1738	
**RT-III-5**	x		x	x	x	1521	In ASC-14; SA03-04 RGP-3; WA40-23C RGP-1; CS36 RGP-3; CS39 RGP-1
**RT-III-6**	x		x	x	x	1053	In ASC-14; SA03-04 RGP-3; WA40-23C RGP-1; CS36 RGP-3; CS39 RGP-1
**RT-III-7**	x		x	x	x	798	In ASC-14; SA03-04 RGP-3; WA40-23C RGP-1; CS36 RGP-3; CS39 RGP-1; LAMP primers designed here [36]
**RT-III-8**			x			1100	
**RT-IV-1**				x		1218	
**RT-IV-2**				x		1120	
**RT-IV-3**				x		1263	In ASC-16; CS36 RGP-4
**RT-IV-4**				x		889	CS36 RGP-4
**RT-IV-5**				x		1270	
**RT-IV-6**				x		1470	
**RT-IV-7**				x		1734	In ASC-17
**RT-IV-8**				x		2731	In ASC-17
**RT-IV-9**				x		1430	
**RT-IV-10**				x		5882	CS36 RGP-6; LAMP primers designed here [36]
**RT-V-1**					x	1725	
**RT-V-2**					x	611	In RT-V unique Thiopeptide Cluster (ASC-18); CS39 RGP-3
**RT-V-3**					x	8451	In RT-V unique Thiopeptide Cluster (ASC-18); CS39 RGP-3; LAMP primers designed here [36]
**RT-V-4**					x	10,447	In RT-V unique Thiopeptide Cluster (ASC-18); CS39 RGP-3

^a^ Region name based on the strain analyzed for unique regions, relative to the others; i.e., RT-III-2 was the second region identified in population RT-III by PanSeq. X denotes which populations the regions were found in by mapping using Geneious.

## Supplementary Materials

The following are available online at https://www.mdpi.com/2076-2607/8/3/366/s1.
